# STRAP and NME1 Mediate the Neurite Growth-Promoting Effects of the Neurotrophic Factor GDF5

**DOI:** 10.1016/j.isci.2020.101457

**Published:** 2020-08-12

**Authors:** Jayanth Anantha, Susan R. Goulding, Sean L. Wyatt, Ruth M. Concannon, Louise M. Collins, Aideen M. Sullivan, Gerard W. O'Keeffe

**Affiliations:** 1 Department of Anatomy & Neuroscience, University College Cork (UCC), Cork, Ireland; 2 Department of Biological Sciences, Cork Institute of Technology, Cork, Ireland; 3 School of Biosciences, Cardiff University, Museum Avenue, Cardiff, UK; 4 Department of Physiology, UCC, Cork, Ireland; 5 APC Microbiome Ireland, UCC, Cork, Ireland; 6 Cork Neuroscience Centre, UCC, Cork, Ireland

**Keywords:** Molecular Biology, Neuroscience, Proteomics

## Abstract

Loss of midbrain dopaminergic (mDA) neurons and their axons is central to Parkinson's disease (PD). Growth differentiation factor (GDF)5 is a potential neurotrophic factor for PD therapy. However, the molecular mediators of its neurotrophic action are unknown. Our proteomics analysis shows that GDF5 increases the expression of serine threonine receptor-associated protein kinase (STRAP) and nucleoside diphosphate kinase (NME)1 in the SH-SY5Y neuronal cell line. GDF5 overexpression increased NME1 expression in adult rat brain *in vivo*. NME and STRAP mRNAs are expressed in developing and adult rodent midbrain. Expression of both STRAP and NME1 is necessary and sufficient for the promotion of neurite growth in SH-SY5Y cells by GDF5. NME1 treatment increased neurite growth in both SH-SY5Y cells and cultured mDA neurons. Expression patterns of NME and STRAP are altered in PD midbrain. NME1 and STRAP are thus key mediators of GDF5's neurotrophic effects, rationalizing their future study as therapeutic targets for PD.

## Highlights

• Mechanism of action of dopaminergic neurotrophic factor GDF5 is not fully understood
• Increased expression of STRAP and NME1 is needed for neurite growth promotion by GDF5
• NME1 increases neurite growth in SH-SY5Y cells and midbrain dopaminergic neurons
• STRAP and NME1 co-expression patterns are altered in Parkinson's disease midbrain

## Graphical Abstract

**Figure:**
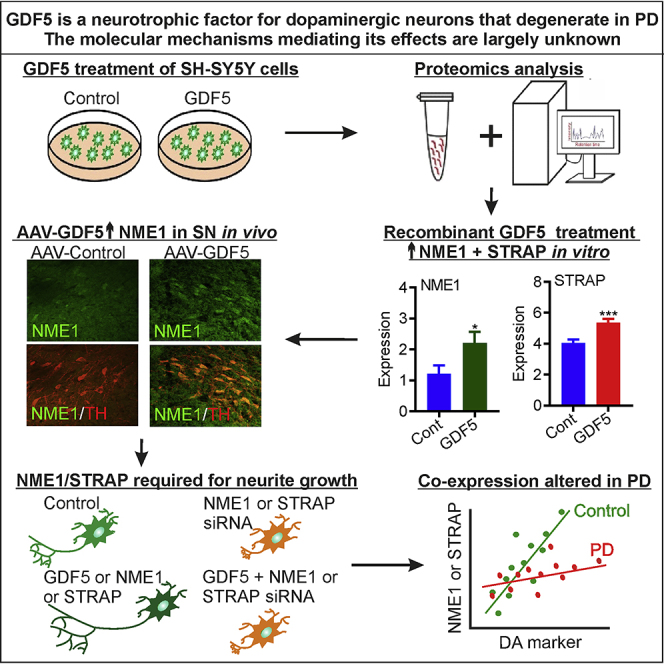

## Introduction

Parkinson's disease (PD) is the second most common neurodegenerative disorder, affecting 1% of the population older than 60 years (Lees et al., 2009; Tysnes and Storstein, 2017). The neuropathological hallmarks of PD include the progressive degeneration of midbrain dopaminergic (mDA) neurons and their axons that project to the striatum via the nigrostriatal pathway (Burke and O'Malley, 2013; Caminiti et al., 2017; O'Keeffe and Sullivan, 2018). The application of neurotrophic factors to halt and potentially reverse the degeneration of the nigrostriatal pathway holds significant promise as a disease-modifying therapy for PD (Kelly et al., 2015; Sullivan and O'Keeffe, 2016). However, despite promising results in open-label clinical trials (Gill et al., 2003; Slevin et al., 2007; Marks et al., 2008), subsequent randomized double-blind trials of the dopaminergic neurotrophic factors, glial cell line-derived neurotrophic factor (GDNF) and neurturin, failed to meet their primary endpoints (Lang et al., 2006; Marks et al., 2010; Warren Olanow et al., 2015; Whone et al., 2019). This has been suggested to be due to the downregulation of Ret, the common co-receptor for GDNF and neurturin, by alpha-synuclein (Decressac et al., 2012), a protein that is present in aggregates, called Lewy bodies, in the PD brain. Additional studies have shown that there is an absolute requirement for Ret for the neurotrophic effect of GDNF on mDA neurons *in vivo* (Drinkut et al., 2016)*.* It is therefore important to characterize the effects of other factors that have neurotrophic effects on mDA neurons, and to decipher the molecular mechanisms that underlie their beneficial effects on this neuronal population.

One such neurotrophic factor is GDF5, a member of the transforming growth factor (TGF)-β superfamily, which was originally identified through its key role in limb development in mice and humans (Krieglstein and Unsicker, 1995; Storm et al., 1994; Storm and Kingsley, 1996). As a member of the TGF-β superfamily, GDF5 is a distant relative of GDNF and has been demonstrated to have similar neuroprotective effects to those of GDNF on mDA neurons *in vitro* (Jaumotte and Zigmond, 2014) and *in vivo* (Sullivan et al., 1998). The neurotrophic and neuroprotective effects of GDF5 have been extensively studied in *in vivo* animal models of PD (Sullivan et al., 1997; 1998; Costello et al., 2012; Hurley et al., 2004). In addition, GDF5 has been shown to promote neurite growth in cultured rat dopaminergic (O'Keeffe et al., 2004a, 2004b; Hegarty et al., 2014) and sympathetic neurons (O’Keeffe et al., 2016), and in the SH-SY5Y human neuroblastoma cell line (Hegarty et al., 2013), which are widely used *in vitro* models of PD. GDF5 has also been reported to enhance neurite complexity, in a hairy enhancer of split (HES)5-dependent manner, in cultured rat hippocampal pyramidal neurons (Osorio et al., 2013).

The neurotrophic effects of GDF5 are exerted through the canonical bone morphogenetic protein (BMP) pathway, which involves signaling through a complex of its two receptors, BMPR1B and BMPR2, resulting in phosphorylation and activation of R-Smad transcription factors, Smad1, Smad5, and Smad9 (Hegarty et al., 2013; Liu et al., 2016). These Smads subsequently form a transcription factor complex with Smad4, which translocates to the nucleus and transcribes target genes (Makkar et al., 2009). This pathway has been shown to mediate the effects of GDF5 in SH-SY5Y cells (Hegarty et al., 2013), cultured rat mDA neurons (Hegarty et al., 2014), and cultured rat sympathetic neurons (O’Keeffe et al., 2016). Silencing of Zinc finger E-box-binding homeobox (ZEB) 2, an inhibitor of the BMP-Smad pathway, resulted in enhanced striatal dopaminergic innervation in cultured rat mDA neurons; furthermore, Zeb2-deficient mice display dopaminergic hyperinnervation of the striatum (Hegarty et al., 2017). This role for the BMP-Smad pathway in striatal dopaminergic innervation is of interest because nigrostriatal axonal degeneration is a key early pathological process in PD (for reviews see Burke and O'Malley, 2013; O'Keeffe and Sullivan, 2018). The fact that GDF5 can exert neurotrophic actions through this Ret-independent pathway is important, given the failure of the Ret-dependent factors, GDNF and neurturin, in clinical trials (Lang et al., 2006; Marks et al., 2010; Warren Olanow et al., 2015; Paul and Sullivan, 2018; Whone et al., 2019). The Ret receptor is thought to be downregulated by alpha-synuclein, which accumulates in the PD brain (Decressac et al., 2012); thus there is a drive to identify and test alternative dopaminergic neurotrophic factors that are not dependent on Ret for signaling. Ret-independent factors that act via receptors and signaling molecules that are not affected by the neuropathology of PD have potential as therapeutic agents.

GDF5 and its receptors are expressed in a temporally and spatially regulated manner in the pre- and postnatal developing embryonic mouse brain, suggesting that GDF5 plays a role in brain development (O'Keeffe et al., 2004b; Hegarty et al., 2014). The expression profiles of GDF5 and GDNF and their receptors differ in hydroxydopamine (6-OHDA) lesion in *in vivo* models of PD. In both striatal and medial forebrain bundle (MFB) lesion models, striatal levels of GDF5 mRNA were increased at 10 days post-lesion, whereas GDNF mRNA levels in the nigrostriatal system were decreased after 10 and 28 days (Gavin et al., 2014). Expression of GDF5's receptors, BMPR1B and BMPR2, was unperturbed in both striatal and MFB-lesioned rats, whereas mRNA levels of the GDNF receptors, Ret and GFR1α, were significantly decreased (Gavin et al., 2014).

Although the protective effects of GDF5 on dopaminergic neurons have been validated *in vitro* and *in vivo*, and the receptors and some of the signaling pathways are known, there is a need for further information regarding the molecular pathways that mediate the effects of GDF5 on mDA neurons, to facilitate the development of targeted therapeutic approaches for PD. To address this, we used the SH-SY5Y cell line to study the effects of GDF5 on the proteome. We initially used SH-SY5Y cells rather than primary dopaminergic neuronal cultures for the proteomics screen, to have a more homogeneous population of cells for analysis. SH-SY5Y cells are widely used in cell biological studies as an *in vitro* model of relevance to PD (Xicoy et al., 2017). Furthermore, GDF5 has similar neurotrophic effects on, and acts via the same pathway in, SH-SY5Y cells as it does in cultured rat mDA neurons (Hegarty et al., 2013, 2014), meaning that this cell line can be used to screen for molecular mechanisms of relevance to mDA neurons.

## Results

### GDF5 Activates Smad Signaling and Induces a Transcriptional Response in SH-SY5Y Cells

**Figure 1:**
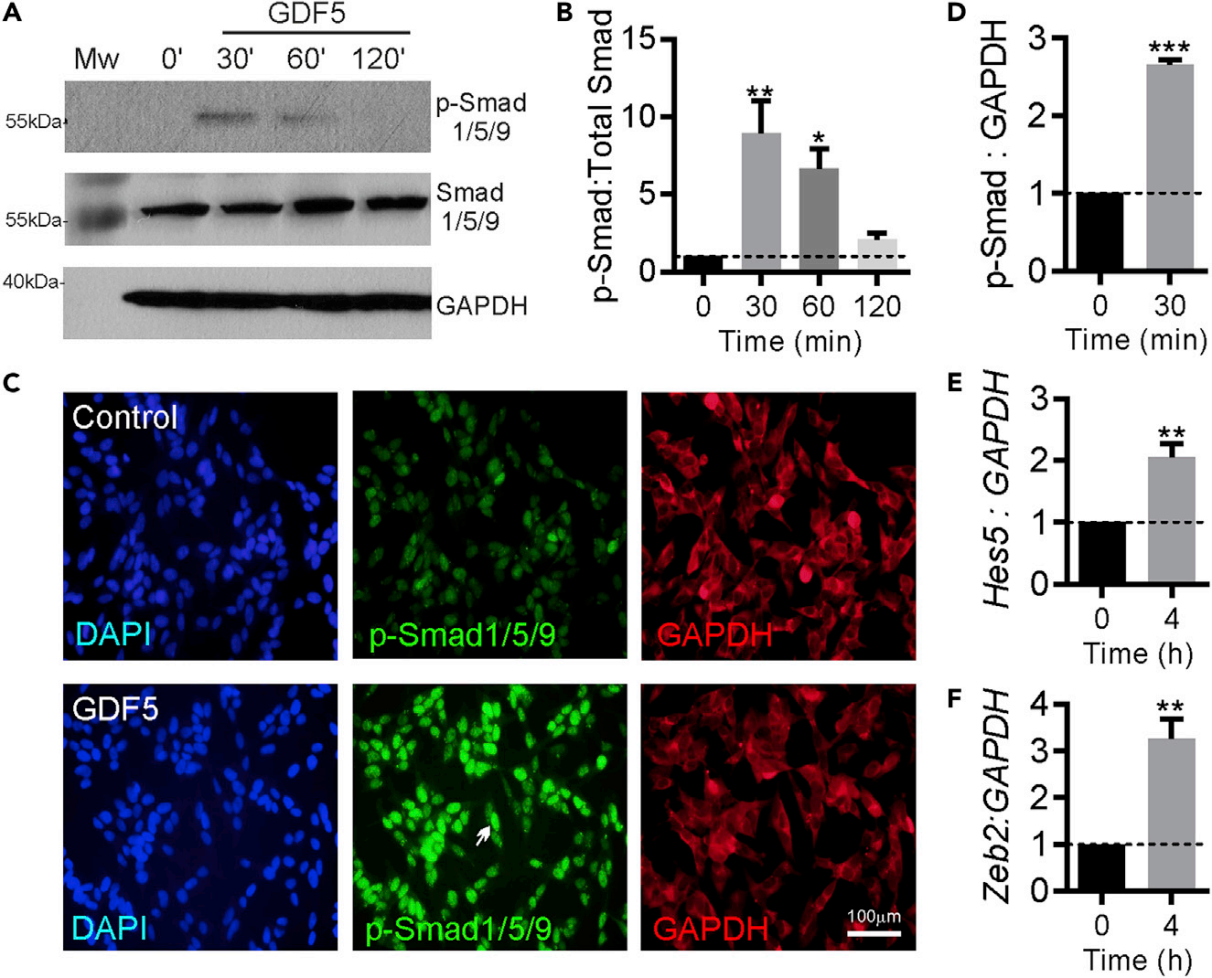
GDF5 Activates Smad Signaling and Induces a Transcriptional Response in SH-SY5Y Cells (A and B) (A) Representative western blots and (B) densitometry showing the relative level of phospho(p)-Smad1/5/9 staining, normalized to that of Smad1/5/9, in SH-SY5Y cells treated with 100 ng/mL GDF5 for 30, 60, or 120 min. Data are mean ± SEM from three independent experiments (*n* = 3) (∗p < 0.05, ∗∗p < 0.01 versus control; one-way ANOVA with Tukey post-hoc test). (C–F) (C) Representative images of p-Smad1/5/9 (green), GAPDH (red), and DAPI (blue) staining and (D) graph showing the relative level of p-Smad1/5/9 staining normalized to that of GAPDH in SH-SY5Y cells treated with 100 ng/mL GDF5 for 30 min. Real-time PCR data showing the relative expression of transcripts for (E) *Hes5* and (F) *Zeb2* mRNA normalized to GAPDH mRNA in SH-SY5Y cells treated with 100 ng/mL GDF5 for 4 h. Data are shown as mean ± SEM from three independent experiments (*n* = 3) (∗∗p < 0.01, ∗∗∗p < 0.001 versus control; Student's t test).

We first confirmed that GDF5 treatment of SH-SY5Y cells activated the canonical BMP-Smad signaling pathway. To do this, SH-SY5Y cells were plated for 24 h before they were treated with 100 ng/mL recombinant human GDF5 for 30, 60, or 120 min. Western blotting revealed a significant increase in the levels of phospho (p)-Smad1/5/9, which peaked at 30 min after treatment (Figures 1A and 1B). We next validated these findings by treating SH-SY5Y cells with GDF5 for 30 min and immunocytochemically staining them for p-Smad1/5/9 and counterstaining with GAPDH and DAPI (Figure 1C). Quantification of the relative nuclear fluorescence intensity revealed a significant increase in the levels of p-Smad1/5/9 after GDF5 treatment (Figure 1D). Finally, to confirm that GDF5-induced increases in p-Smad1/5/9 translated to a transcriptional response, we examined the expression of transcripts for the *Hes5* and *Zeb2,* which are known downstream targets of the BMP-Smad pathway. Real-time PCR confirmed that a 240-min treatment with 100 ng/mL GDF5 led to a significant increase in the expression of *Hes5* and *Zeb2* mRNA (Figures 1E and 1F). Collectively, these data show that GDF5 activates canonical BMP-Smad signaling in SH-SY5Y cells, making them a suitable model for a proteomics screen to identify novel mediators of the effects of GDF5.

### GDF5-Induced Changes in the Proteome in SH-SY5Y Cells and Gene Co-expression Analysis Using Data from the Human Substantia Nigra (SN) Identifies Correlated Patterns of STRAP and NME1 Expression

**Figure 2:**
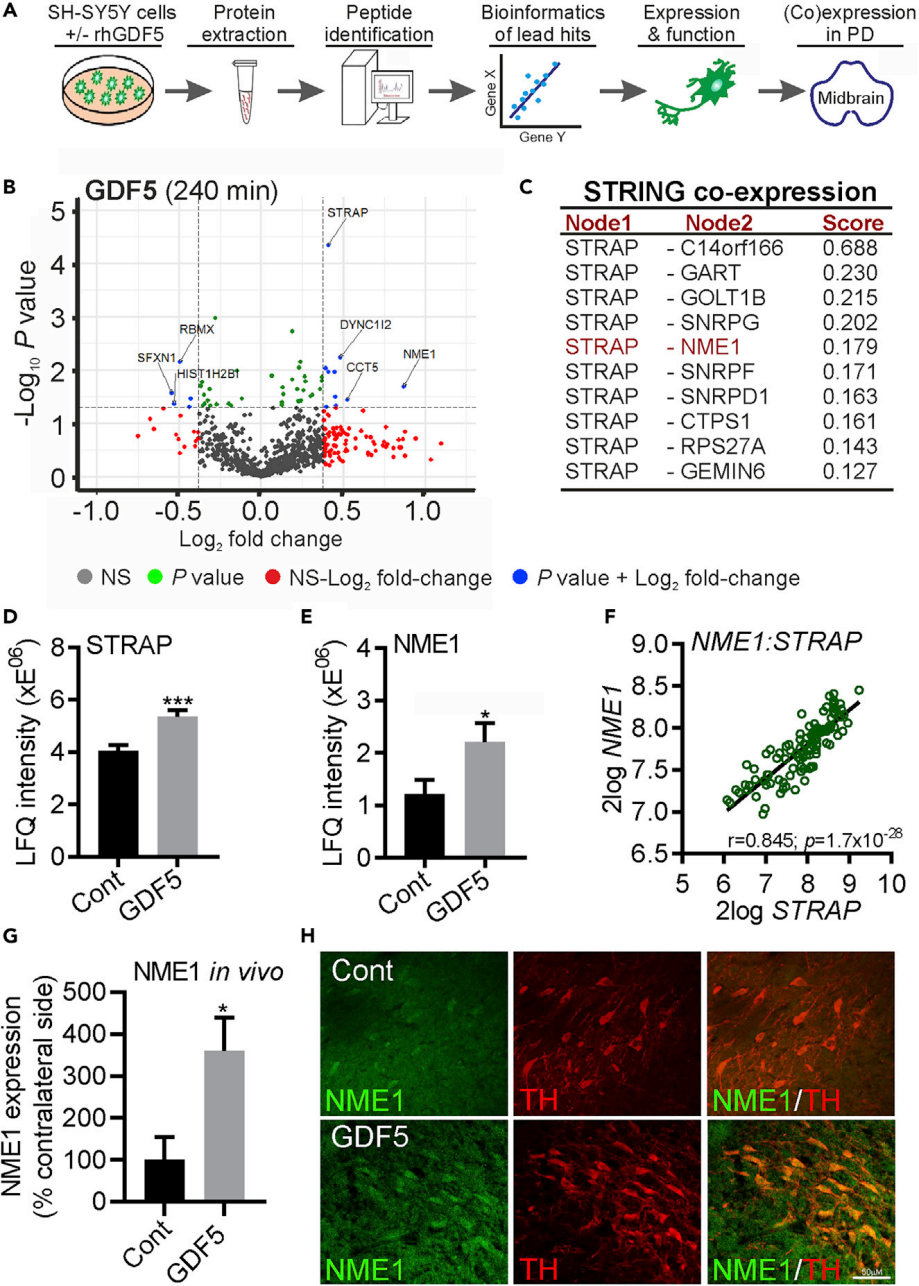
GDF5-Induced Changes in the Proteome in SH-SY5Y Cells and Gene Co-expression Analysis of Data from the Human SN Identifies Correlated Patterns of STRAP and NME1 Expression (A) Schema showing the experimental workflow using to identify changes in the proteome induced by GDF5 in SH-SY5Y cells. (B) Volcano plots of the -log_10_p values and the log_2_Fold change of proteins that were altered in SH-SY5Y cells following treatment with 100 ng/mL GDF5 for 240 min. (C–E) (C) Table showing the co-expression scores for genes associated with STRAP in STRING with a medium confidence threshold, which identified NME1 (highlighted in red), which was also found in the proteomic analyses. Graphs showing the LFQ intensities from the proteomics screen at 240 min for (D) STRAP and (E) NME1. Data are mean ± SEM from three independent samples per group (*n* = 3) (∗p < 0.05, ∗∗∗p < 0.001 versus control; Student's t test). (F) Linear regression showing the correlation between *STRAP* and *NME1* in the human SN (*n* = 101). The *r* and Bonferroni-corrected p values are shown on the graph. All transcriptome data are 2log expression values. Raw data was obtained from the Gene expression Omnibus GSE60863 and analyzed using the R2: Genomics analysis and visualization platform (https://hgserver1.amc.nl/cgi-bin/r2/main.cgi). (G) Graph showing NME1 expression in the SN of adult rats that had received AAV-Control (“Cont”) or AAV-GDF5 (“GDF5”), as a percentage of that on the contralateral side. Data are mean ± SEM from *n* = 4 rats (∗p < 0.05 versus control; unpaired Student's t test). (H) Representative photomicrograph of NME1 (green) and TH (red) expression in the SN of AAV-Cont and AAV-GDF5 animals. Scale bar, 50 μm.

To identify novel regulators of the neurotrophic effects of GDF5, we carried out an untargeted proteomics analysis of human SH-SY5Y cells treated with GDF5 (Figure 2A). SH-SY5Y cells were treated with 100 ng/mL GDF5 for 240 min. Total proteins were then extracted from cell lysates and analyzed using liquid chromatography-tandem mass spectrometry (LC-MS/MS). The resulting peptides were identified using MaxQuant (version 1.6.0.16). A false discovery rate (FDR) of 1% was used to identify unique peptides mapping to a protein. The resulting label-free quantification (LFQ) values were used to calculate the fold change and p values. The -log_10_p value and the log_2_Fold change were calculated and plotted in a volcano plot using R-package enhanced volcano from Bioconductor. The resulting volcano plot identified 10 proteins with a fold change ≥1.3 and a p value ≤0.05, and identified 5 proteins with a fold change ≤0.7 and a p value ≤ 0.05 (Figure 2B). We then examined this list of differentially expressed proteins for those that might be candidates to regulate BMP-Smad signaling. Among this list was a protein called serine-threonine kinase receptor protein (STRAP; UniProtKB Q9Y3F4). This protein was selected for further study as the BMPRs are serine-threonine kinase receptors, and STRAP has been shown to negatively regulate TGF-β signaling and to positively regulate protein 3-phosphoinositide-dependent protein kinase-1 (PDPK1) kinase activity by directly binding to it (Seong et al., 2005). We next identified proteins that were associated with STRAP by generating a protein-protein interaction network using STRING (https://string-db.org/) with STRAP as the hub protein. This analysis identified 29 proteins including PDPK1 (Seong et al., 2005), which confirmed the sensitivity of the approach. We then screened this list and found among it the protein nucleoside diphosphate kinase A (NME1; UniProtKB: P15531) (Figure 2C), which was also upregulated by GDF5 (Figure 2B). We next plotted the LFQ intensities of STRAP and NME1 from the LC-MS/MS proteomics data. This showed that the expression of STRAP (Figure 2D) and NME1 (Figure 2E) was significantly increased at 240 min after GDF5 treatment. We next used gene co-expression analyses, which is a recently developed approach for the analysis of cellular function based on correlated patterns of gene expression that reflect potential functional associations (Eisen et al., 1998; Homouz and Kudlicki, 2013). If STRAP and NME1 have a functional association in mDA neurons, we would expect them to display a gene co-expression pattern in the midbrain *in vivo*. To test this, we used available gene expression data (Gene expression Omnibus: GSE60863; Ramasamy et al., 2014), to examine whether the expression of NME1 and STRAP had a significant positive correlation in the human substantia nigra (SN). In agreement with the STRING analysis, we found that STRAP and NME1 displayed a strong positive correlation in the human SN (*r* = 0.845; *p =* 1.7 × 10^−28^) (Figure 2F). Collectively, these data suggest a GDF5-STRAP-NME1 functional association. We also performed immunohistochemical staining on sections of adult rat brain that had been transduced with adeno-associated virus (AAV)-control or AAV-GDF5 and found increased levels of NME1 protein in dopaminergic (tyrosine hydroxylase [TH]-immunopositive) neurons within the SN of rats that had received AAV-GDF5, compared with controls (Figures 2G and 2H). This confirmed that GDF5 overexpression upregulates NME1 *in vivo.*

### *Strap* and *Nme1* Are Expressed in the Developing and Adult Rodent Midbrain

**Figure 3:**
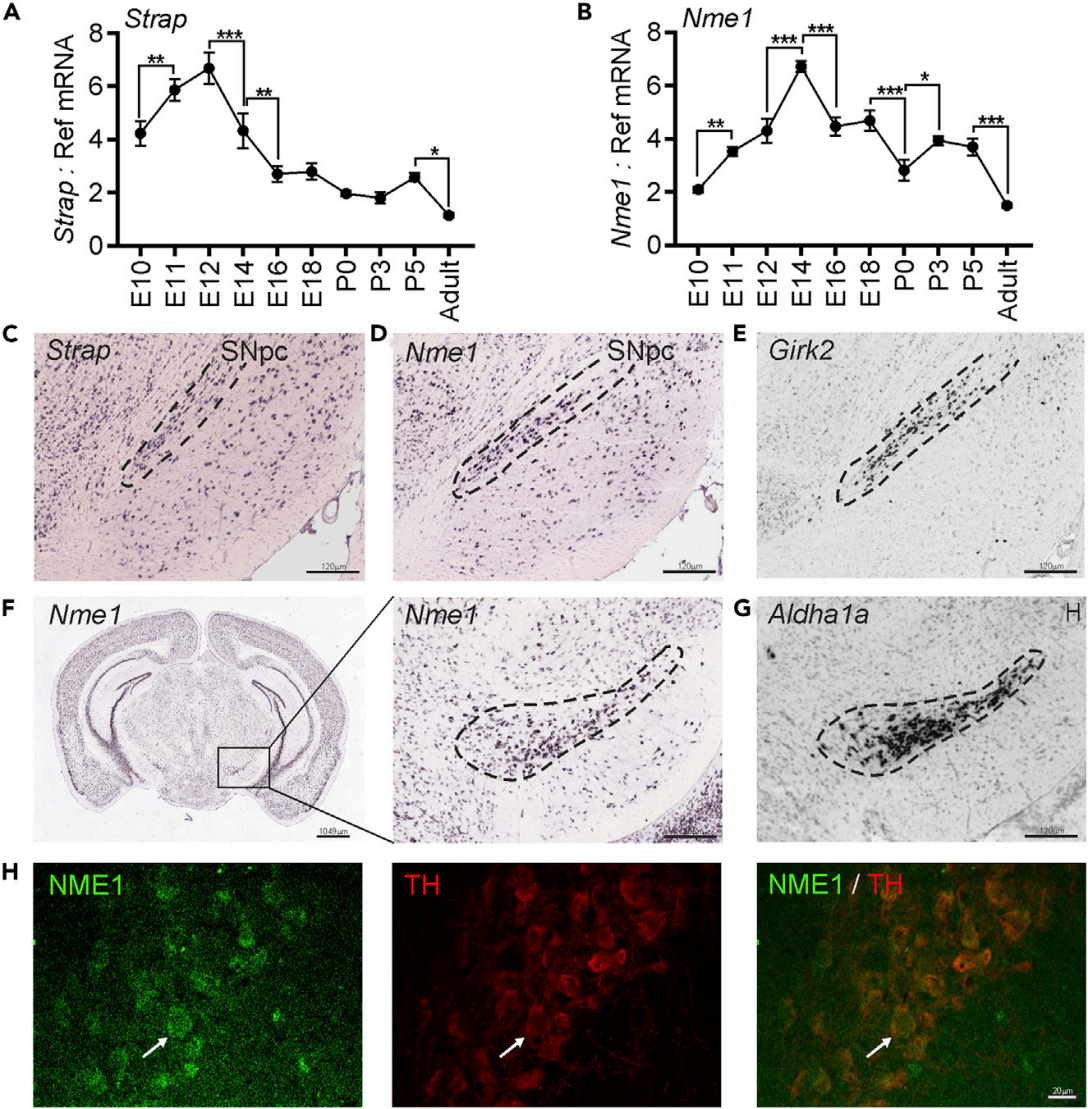
*Strap* and *Nme1* Are Expressed in the Developing and Adult Rodent Midbrain (A–G) RT-qPCR showing the expression of transcripts for (A) *Strap* and (B) *Nme1* in mouse midbrain from embryonic day (E) 10 to postnatal day (P) 90 (adult) relative to the levels of the geometric mean of three reference mRNAs, *Gapdh*, *Sdha,* and *Hprt1*. Data are mean ± SEM from *n* = 4 mice at each time point. (∗p < 0.05, ∗∗p < 0.01, ∗∗∗p < 0.01 as indicated; one-way ANOVA with *post-hoc* Fisher's least significant difference (LSD) test, comparing each time point to the one immediately before and after it). Images of sagittal sections of the P56 mouse brain showing the expression of transcripts for (C) *Strap* and (D) *Nme1* compared with that of (E) *Girk2*. Images of coronal sections of the P56 mouse brain showing the expression of (F) *Nme1* in the (G) *Aldh1a1* domain. The dashed lines indicate the SNpc. Image credit: Allen Institute. (H) Representative confocal images of the adult rat SN immunofluorescently stained for NME1 (green) and TH (red). Scale bar, 20 μm.

We next sought to determine whether Strap and Nme1 were expressed in the mouse midbrain *in vivo.* To do this, we quantified the levels of *Strap* and *Nme1* mRNAs in the mouse ventral midbrain (VM) at intervals throughout the period of initial mDA differentiation, axon growth, and striatal innervation, and in adulthood, using quantitative real-time PCR. *Strap* and *Nme1* displayed somewhat similar patterns of expression throughout development. *Post-hoc* testing showed that the expression of these mRNAs increased significantly from E10, and that *Strap* was maximally expressed at E12 (Figure 3A), whereas *Nme1* was maximally expressed at E14 (Figure 3B). The expression levels of both mRNAs subsequently decreased from this point in development onward (Figures 3A and 3B). There was also a significant decrease in the expression of *Strap* and *Nme1* from P5 to P90 (Figures 3A and 3B). *In situ* hybridization data from the Allen Mouse Brain Atlas confirmed the co-localization of *Strap* (Figure 3C) and *Nme1* (Figures 3D and 3F) transcripts with the expression of the mDA markers, *Girk2* and *Aldh1a1* (Figures 3E and 3G), in adult mouse substantia nigra pars compacta (SNpc). To verify that these proteins are co-expressed in adult rat SN, we performed immunohistochemical staining, which showed that NME1 was expressed in adult rat SN, in all TH-immunopositive neurons (Figure 3H). This confirms the expression of NME1 in adult mDA neurons.

### Gene Co-expression Analysis of Human SN Identifies *STRAP* and *NME1* Co-expression with Multiple Markers of Midbrain Dopaminergic Neurons

**Figure 4:**
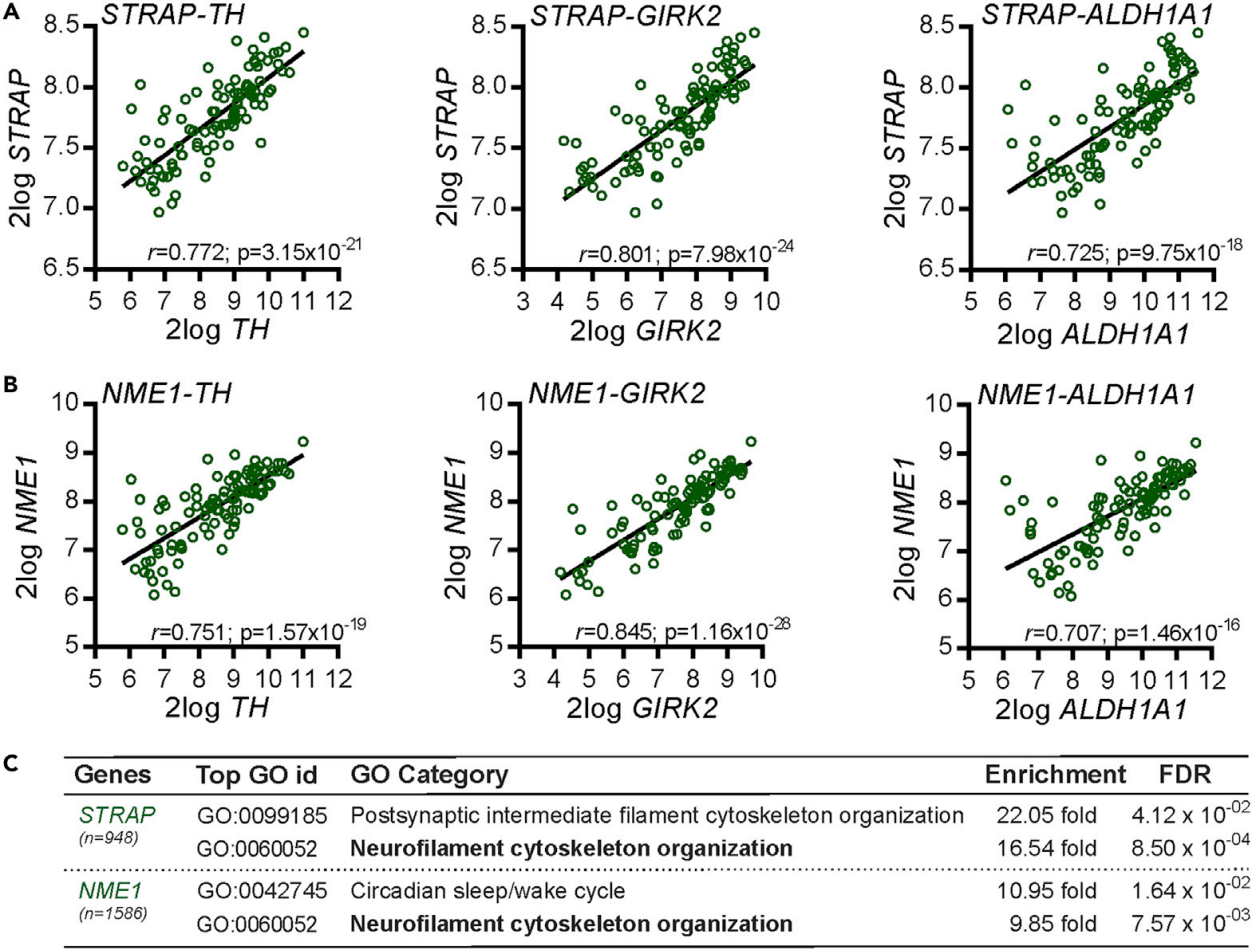
Gene Co-expression Analysis of Human SN Identifies *STRAP* and *NME1* Co-expression with Multiple Markers of Midbrain Dopaminergic Neurons (A and B) Linear regression showing the correlation between (A) *STRAP* and (B) *NME1* and three markers of midbrain dopaminergic neurons (*TH, GIRK2, ALDH1A1*) in the human SN (n = 101). The *r* and Bonferroni-corrected p values are shown on each graph. Raw data were derived from dataset GSE60863 from the Gene Expression Omnibus and analyzed using the R2 microarray platform. (C) Table showing the number of genes in the human SN displaying a multiple testing-adjusted correlation of ≥0.7 with *STRAP* (*n* = 948) and *NME1* (*n* = 1,586), along with a GO enrichment analysis of these gene lists. The top two GO categories based on fold-enrichment in each gene list are shown, along with the fold-enrichment and false discovery rate (FDR)-adjusted p value.

Building on the analyses of the mouse brain, we next investigated whether *STRAP* and *NME1* were co-expressed with multiple markers of mDA neurons in the human SN. To investigate this, we again used available gene expression data from the Gene expression Omnibus (GSE60863) to determine if *STRAP* and *NME1* displayed any significant positive correlation with the expression of three markers of mDA neurons: *TH,* G-protein-regulated inward rectifier potassium channel (*GIRK*) 2, and aldehyde dehydrogenase (*ALDH*) *1A1.* Both STRAP and *NME1* were found to have a significant positive correlation with all three mDA markers. These data show that transcripts for *STRAP* (Figure 4A) and *NME1* (Figure 4B) display a positive co-expression pattern with transcripts for multiple markers of mDA neurons in the human SN, suggesting that they may play a functional role in mDA neurons. To gain some insight into this, we next identified all genes that had a significant positive correlation with transcripts for *STRAP* and *NME1* in the human SN (Figure 4C). A gene ontology (GO) enrichment analysis revealed that the GO category “*Neurofilament cytoskeletal organisation*” (Figure 4C) was among the top two GO categories overrepresented in the lists of genes that displayed a co-expression pattern with *STRAP* and *NME1*. As “*Neurofilament cytoskeletal organisation*” was the common ontology of both STRAP and NME1, we examined the co-expression of NME1 and STRAP with all genes annotated for this ontology in the human SN. The results showed significant positive correlation between the expression of NME1 and STRAP with Internexin neuronal intermediate filament protein alpha (INA), Neurofilament heavy (NEFH), ATPase phospholipid transporting 8A2 (ATP8A2), Neurofilament light (NEFL), superoxide dismutase 1(SOD1), and NudE neurodevelopment protein 1 like 1(NEDL1) (Table 1). Collectively, these data show that *STRAP* and *NME1* are expressed in the mouse and human SN, and suggest that *STRAP* and *NME1* may regulate neurite growth and/or the neurite growth-promoting effects of GDF5.

**Table 1:** List of Genes Annotated for GO:0060052 (Neurofilament Cytoskeletal Organisation) which Are Co-expressed with NME1 and STRAP

Ensemble Gene ID	Gene Symbol	Description	Co-expression with NME1	Co-expression with STRAP
ENSG00000148798	INA	*Internexin neuron intermediate filament protein alpha*	*r* = 0.892 p = 5.12 × 10^−32^	*r* = 0.719 p = 2.12 × 10^−13^
ENSG00000100285	NEFH	*Neurofilament heavy*	*r* = 0.862 p = 4.39 × 10^−27^	*r* = 0.798 p = 1.47 × 10^−19^
ENSG00000132932	ATP8A2	*ATPase phospholipid transporting 8A2*	*r* = 0.872 p = 1.54 × 10^−28^	*r* = 0.760 p = 2.68 × 10^−16^
ENSG00000277586	NEFL	*Neurofilament light*	*r* = 0.890 p = 1.60 × 10^−31^	*r* = 0.740 p = 8.14 × 10^−15^
ENSG00000142168	SOD1	*Superoxide dismutase 1*	*r* = 0.909 p = 1.53 × 10^−35^	*r* = 0.850 p = 2.32 × 10^−25^
ENSG00000166579	NDEL1	*NudE neurodevelopment protein 1 like 1*	*r* = 0.895 p = 1.71 × 10^−32^	*r* = 0.748 p = 2.21 × 10^−15^

### STRAP and NME1 Are Necessary for Basal and GDF-5-Induced Neurite Growth in SH-SY5Y Cells

**Figure 5:**
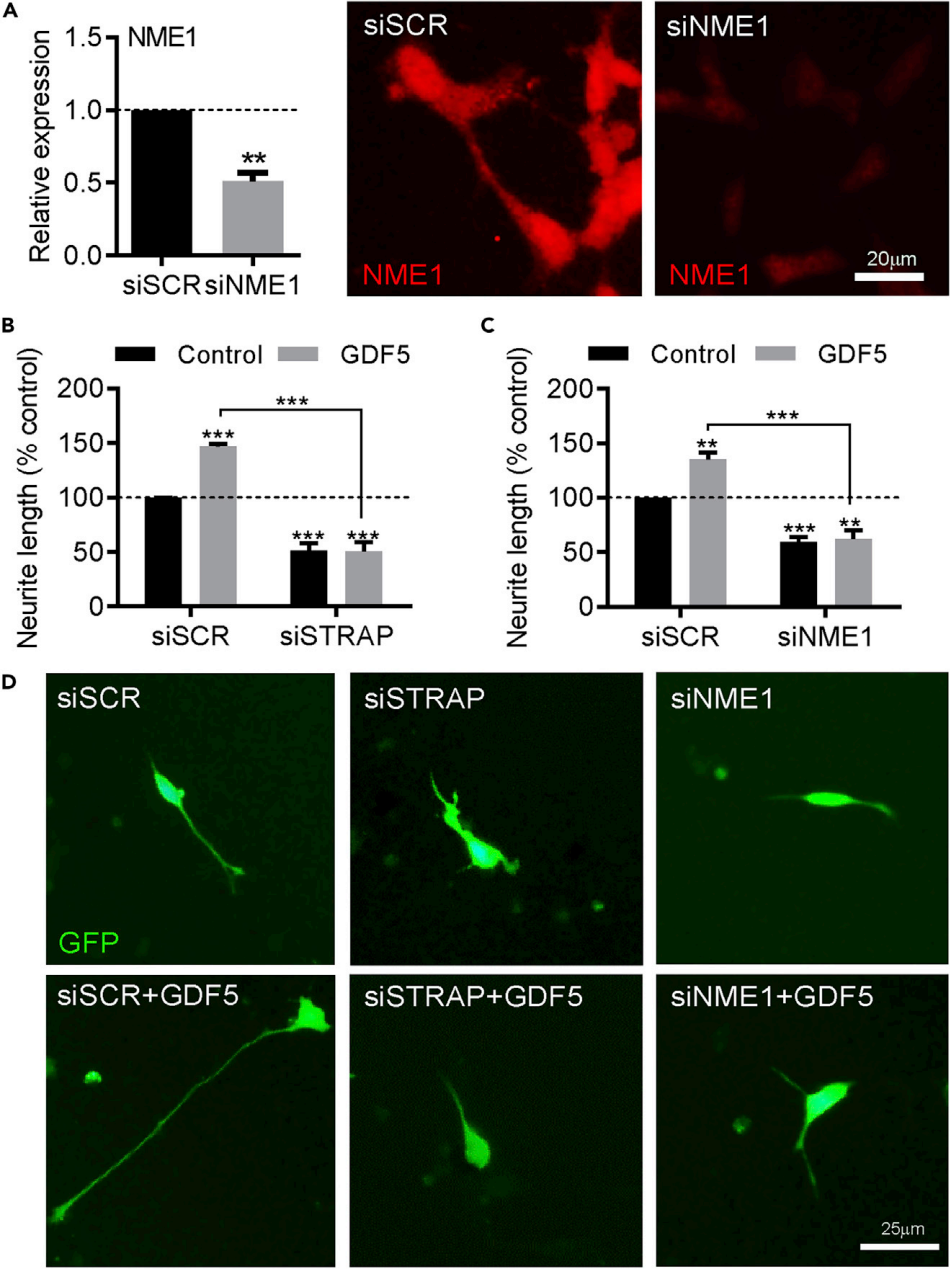
STRAP and NME1 Are Necessary for Basal and GDF-5-Induced Neurite Growth in SH-SY5Y Cells (A) Graph and representative photomicrographs showing NME1 expression in SH-SY5Y cells transfected with scrambled siRNA (siSCR) or NME1 siRNA (siNME1). (B–D) (B and C) Graphs of neurite length as percentage of control, and (D) representative photomicrographs of SH-SY5Y cells transfected with siSCR or (B and D) siRNA targeting STRAP (siSTRAP) or (C and D) siNME1 and cultured with or without 100 ng/mL GDF5 for 72 h. Data are mean ± SEM from three independent experiments (*n* = 3) (∗∗p < 0.01, ∗∗∗p < 0.001 versus siSCR control or as indicated; two-way ANOVA with *post-hoc* Fisher's LSD test or Student's t test as appropriate).

To test the above-mentioned hypothesis, we next used neurite outgrowth as a phenotypic readout to examine the effect of small interfering RNAs (siRNAs) against *STRAP* and *NME1* in individual cells. To do this, we transfected SH-SY5Y cells with 25 nM of a scrambled siRNA (siSCR) or siRNAs targeting *STRAP* (siSTRAP) or *NME1* (siNME1) together with a GFP expression plasmid to identify transfected cells, which led to a significant reduction in cellular NME1 expression (Figure 5A). Following transfection, the cells were cultured with or without 100 ng/mL GDF5 and neurite growth was analyzed at 72 h. Transfection with siSTRAP led to a significant reduction in neurite growth compared with siSCR control (51.5% ± 6.5% of the siSCR control) (Figure 5B). GDF5 treatment led to a significant increase in neurite growth in cells transfected with the siSCR (p < 0.001; 135.1% ± 6.7% of the control) (Figure 5B). In contrast, neurite growth in cells transfected with siSTRAP and cultured with GDF5 was significantly reduced compared with siSCR alone (p < 0.01), and siSTRAP completely prevented the neurite growth-promoting effects of GDF5 (siSTRAP: 51.5% ± 6.5% versus siSTRAP + GDF5: 50.7% ± 8.5%) (Figure 5B). Similarly, transfection with siNME1 led to a significant reduction in neurite growth compared with siSCR control (59.5% ± 1.3% compared with the siSCR control) (Figure 5C). Treatment with GDF5 led to a significant increase in neurite growth in cells transfected with the siSCR (p < 0.01; 135.1% ± 6.7% of the control). In contrast, neurite growth in cells transfected with siNME1 and cultured with GDF5 was significantly reduced compared with the siSCR group, and siNME1 completely prevented the effects of GDF5 on neurite growth (siNME1: 59.5% ± 1.3% versus siNME1 + GDF5: 62.1% ± 7.9%) (Figure 5C).

### Overexpression of STRAP or NME1 Is Sufficient to Promote Neurite Growth in SH-SY5Y Cells

**Figure 6:**
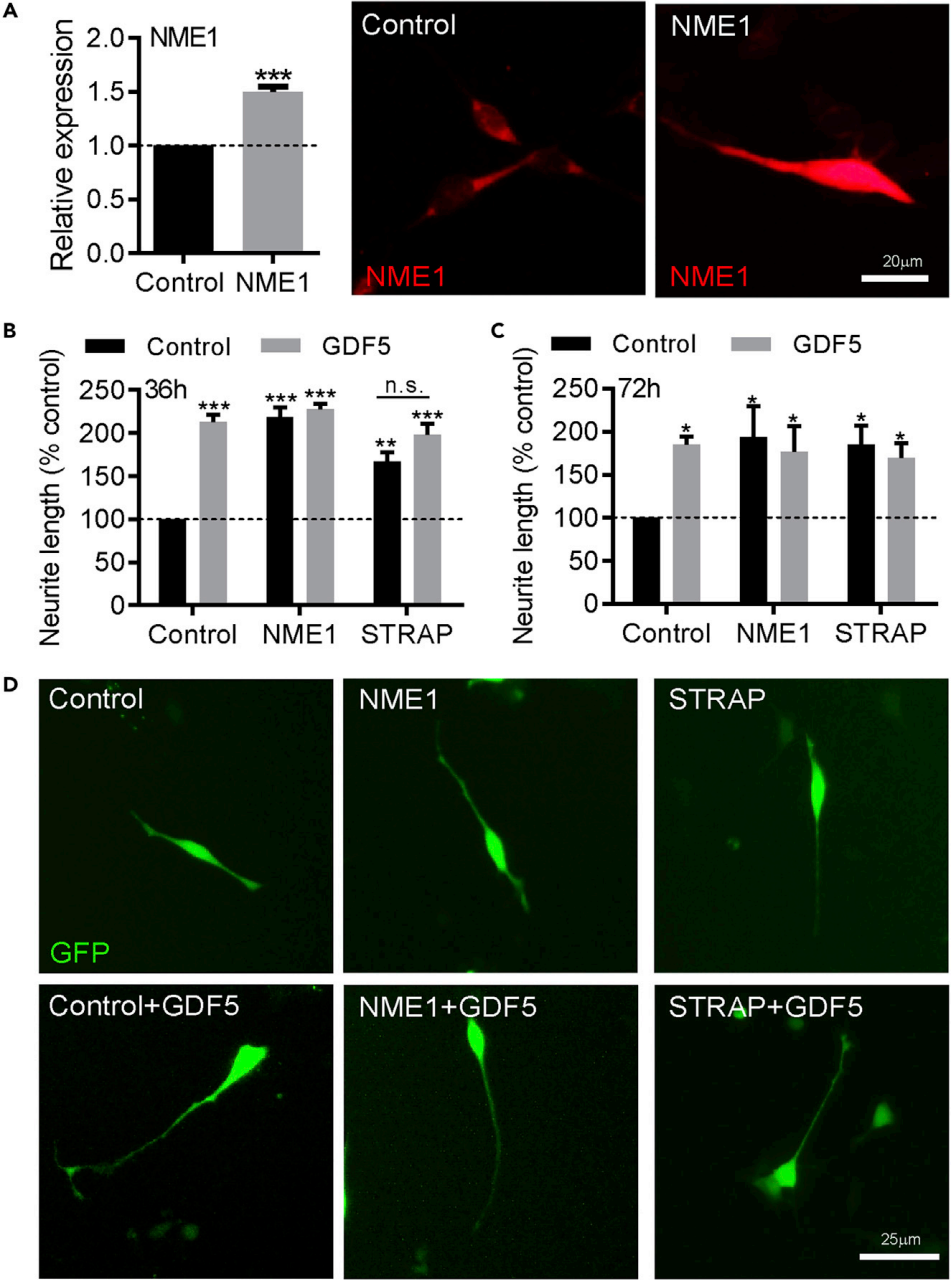
Overexpression of STRAP or NME1 Is Sufficient to Promote Neurite Growth in SH-SY5Y Cells (A) Graph and representative photomicrographs showing NME1 expression in SH-SY5Y cells transfected with control or NME1 expression plasmid. (B–D) (B and C) Graphs of neurite length as percentages of control and (D) representative photomicrographs of SH-SY5Y cells transfected with plasmids overexpressing NME1 or STRAP and cultured with or without 100 ng/mL GDF5 for (B) 36 h or (C) 72 h, as indicated. Data are mean ± SEM from three independent experiments (*n* = 3) (∗p < 0.05, ∗∗p < 0.01, ∗∗∗p < 0.001 versus siSCR control or as indicated. n.s. = not significant; two-way ANOVA with *post-hoc* Fisher's LSD test or Student's *t* test where appropriate).

Given that siRNA targeting NME1 or STRAP reduced neurite growth, we next examined whether NME1 and STRAP overexpression could promote neurite growth. To do this, we transfected SH-SY5Y cells with plasmids overexpressing Myc-tagged NME1 or STRAP, while a GFP-expressing plasmid was used as a control. This resulted in significant overexpression of NME1 protein (Figure 6A). Following transfection, the cells were cultured with or without 100 ng/mL GDF5 and neurite growth was analyzed at 36 h post-transfection. NME1 overexpression led to a significant increase in neurite growth at 36 h (218.5% ± 11.5% versus control), with or without GDF5 (Figure 6B). Similarly, overexpression of STRAP led to a significant increase in neurite growth (166.7% ± 11.1% versus control) with or without GDF5 (Figure 6B). The ability of overexpressed NME1 or STRAP to promote neurite growth persisted to 72 h in culture (Figure 6C). Collectively, these data show that STRAP and NME1 are both necessary and sufficient for basal and GDF5-promoted neurite growth.

### Recombinant NME1 Increases Neurite Growth in SHSY5Y Cells and in Cultured E14 Rat mDA Neurons

**Figure 7:**
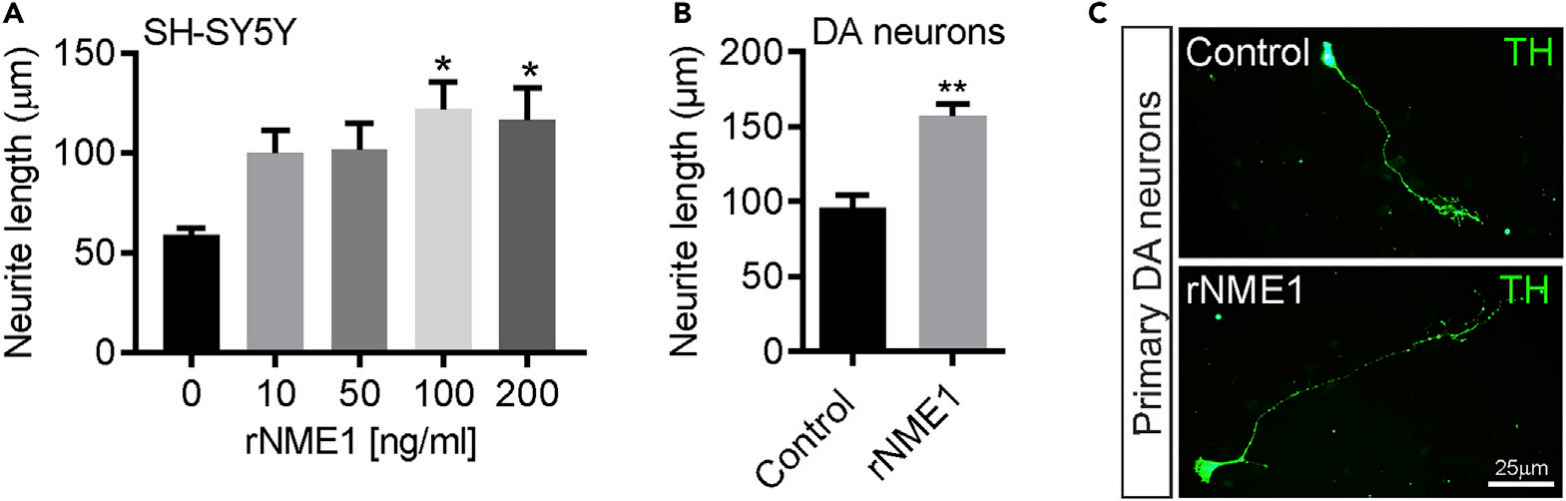
Recombinant NME1 Increases Neurite Growth in SHSY5Y Cells and in Cultured E14 rat mDA Neurons (A and B) Graphs showing neurite lengths of (A) SHSY5Y cells after treatment for 48 h with 0, 10, 50, 100, or 200 ng/mL recombinant human NME1 and (B) E14 mDA neurons after treatment for 48 h with 100 ng/mL recombinant human NME1. (C) Representative photomicrographs of cultured E14 mDA neurons after treatment for 48 h with 0 or 100 ng/mL recombinant NME1, immunocytochemically stained for TH (green). Data are mean ± SEM from three independent experiments (*n* = 3) (∗p < 0.05, ∗∗p < 0.01 versus control; one-way ANOVA with *post-hoc* Fisher's LSD test or Student's t test where appropriate).

Overexpression of NME1 was found to significantly increase neurite length in SH-SY5Y cells. We further tested whether treatment of SH-SY5Y cells and cultured E14 rat mDA neurons with recombinant human NME1 would also increase neurite length. Treatment of SH-SH5Y cells for 48 h with 100 or 200 ng/mL recombinant NME1 resulted in significant increases in neurite length (Figure 7A), as did treatment for 48 h of cultured E14 mDA neurons with 100 ng/mL recombinant NME1 (Figures 7B and 7C).

### Alterations in the Expression of STRAP and NME1 in the PD SN

**Figure 8:**
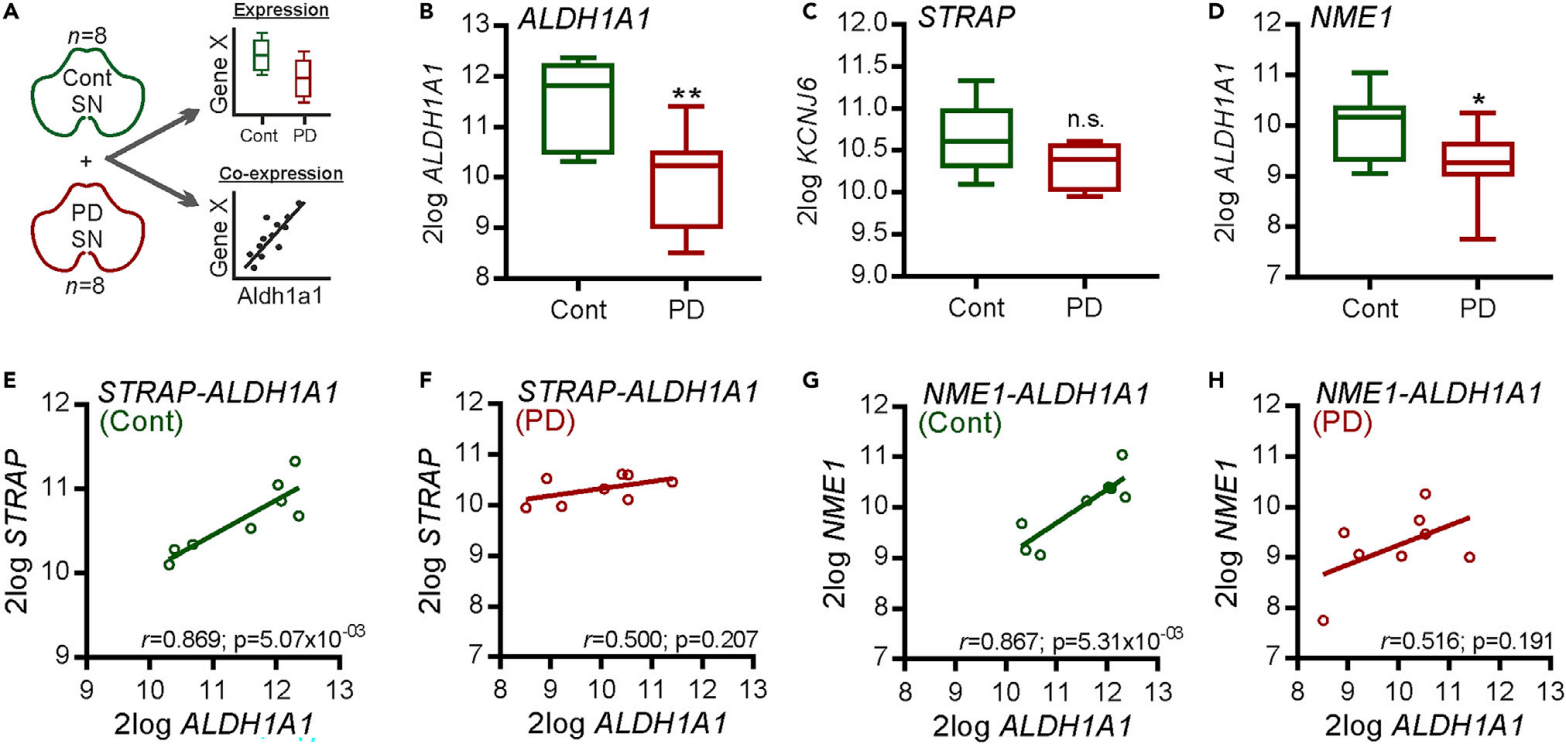
Alterations in the Expression of STRAP and NME1 in the PD SN (A) Schema showing the experimental approach. Raw data were derived from dataset GSE49036, and the R2 microarray platform was used to analyze the expression and co-expression of *STRAP* and *NME1* in PD. (B–D) Boxplots showing the log2 expression of (B) ALDH1A1, (C) STRAP, and (D) NME1 in control (Cont) and PD SN samples (∗p < 0.05, ∗∗p < 0.01 versus control; Student's t test). (E–H) Linear regression analysis showing correlations between (E and F) *STRAP* and *ALDH1A1* and (G and H) *NME1* and *ALDH1A1* in (E and G) control and (F and H) PD samples. The *r* and Bonferroni-corrected p values are shown on the graph.

Finally, we analyzed data from age- and gender-matched samples available in the Gene Expression Omnibus: GSE49036 (Dijkstra et al., 2015) to examine *NME1* and *STRAP* expression, as well as co-expression patterns in the SN, of patients with PD at Braak stage 5/6 (*n* = 8), and of control subjects (*n* = 8) (Figure 8A). As expected, there was significant lower expression of the mDA neuron marker *ALDH1A1* in PD samples compared with controls, which validated the approach (Figure 8B). Although there was a trend toward a decrease in *STRAP* expression in PD samples, this did not reach statistical significance (Figure 8C). However, there was a statistically significant downregulation of *NME1* in the SN of patients with PD compared with controls (Figure 8D). We also examined the co-expression pattern of *STRAP* and *NME1* with *ALDH1A1* in these PD and control SN samples. The rationale for doing this is that in a range of diseases, normal co-expression patterns tend to break down, and these broken correlations can be used as an index of functional misregulation (Torkamani et al., 2010; Zhang et al., 2013; Southworth et al., 2009). In agreement with our earlier analyses, we found that both *STRAP* (*r* = 0.869, p = 5.07 × 10^−3^) (Figure 8E) and *NME1* (*r* = 0.867, p = 5.31 × 10^−3^) (Figure 8G) showed significant positive correlation with *ALDH1A1* in the controls. In contrast, there was no positive correlation of both *STRAP* (*r* = 0.500, p = 0.207) and *NME1* (*r* = 0.516, p = 0.191) with *ALDH1A1* in PD SN samples (Figures 8F and 8H). These broken correlations suggest a functional misregulation of *STRAP-NME1* in PD.

## Discussion

GDF5 is a neurotrophic factor that has been shown to protect mDA neurons from neurotoxic insults *in vitro* and *in vivo* (Sullivan et al., 1997, 1998; Hurley et al., 2004, Toulouse et al., 2012; Costello et al., 2012). In addition, it is well established that GDF5 promotes neurite growth in SH-SY5Y cells and in cultured rodent mDA neurons (O'Keeffe et al., 2004a, 2004b; Hegarty et al., 2013, 2014), enhances neurite complexity in cultured murine hippocampal neurons (Osorio et al., 2013), and increases axonal growth in mouse superior cervical ganglial cells *in vitro* (O’Keeffe et al., 2016). The effects of GDF5 on mDA neurons are dependent on BMPR1B and Smad signaling (Hegarty et al., 2014; Hegarty et al., 2018), whereas the downstream changes that mediate the neurotrophic effects of GDF5 on mDA neurons are unknown. This is important because GDF5 has been proposed as a candidate neurotrophic factor for therapeutic application in PD (O’Keeffe et al., 2017; Paul and Sullivan, 2018), where a characteristic feature is axonal degeneration (Burke and O'Malley, 2013; O'Keeffe and Sullivan, 2018).

In neuronal populations other than mDA neurons, it has been demonstrated that GDF5 can regulate dendritic size and complexity through the upregulation and recruitment of HES5 transcription factor (Osorio et al., 2013). That study also showed that, during development, GDF5 null mice displayed impairments in dendritic growth from hippocampal pyramidal cells (Osorio et al., 2013). Another study, which examined the role of Dachshund family transcription factor (DACH) 2-histone deacetylase (HDAC) 9 signaling in re-innervation of muscle endplates, showed that HDAC9 regulates GDF5 expression to enhance motor functioning by promoting innervation in disrupted neuromuscular junctions (Macpherson et al., 2015). In the present study, in agreement with past work from our group (Hegarty et al., 2013), we show that in the SH-SY5Y cell line, GDF5 activates the BMP-Smad pathway by phosphorylation of Smad1/5/9 and its subsequent nuclear localization. We also found that, similar to the situation in cultured hippocampal neurons (Osorio et al., 2013), GDF5 treatment results in upregulation of *Hes5* and *Zeb2* mRNA in SH-SY5Y cells. We used this model to perform proteomic analysis, investigating the downstream changes in protein expression that are required for the effects of GDF5 on neurite growth in SH-SY5Y cells. This analysis revealed significant upregulation of two proteins, known as STRAP and NME1, in GDF5-treated cells. Furthermore, we examined the SN from the brains of adult rats that had received intranigral AAV-GDF5 and found increased levels of NME1 protein compared with controls. This establishes that GDF5-mediated increases in NME1 protein levels occur not only in SH-SH5Y cells but also are found in the adult rat brain *in vivo.*

NME1 is a protein that possesses serine/threonine-specific protein kinase activity and plays a role in neural development; it has been reported to be downregulated in the SN of patients with PD, in a study of genes related to purine metabolism (Garcia-Esparcia et al., 2015). NME1 is also known to physically interact with STRAP and to negatively regulate TGFβ-mediated signaling (Seong et al., 2007). We used gene co-expression analysis to show that *STRAP* and *NME1* had a strong co-expression pattern in the human SN, which suggests a functional association between these two proteins. STRAP was initially isolated from HeLa cells, as a component of survival of motor neuron complex (SMN) (Meister et al., 2001), and is known to co-localize with SMN complex components in neurite projections formed in nerve growth factor (NGF)-treated PC12 cells (Sharma et al., 2005). NME1 has been reported to play several roles in neuronal growth, and it is known to play a role in fate determination of glial progenitor cells, directing them toward a neuronal fate (Owlanj et al., 2012). These findings suggest roles for NME1 and STRAP in neuronal growth and development. In support of this, we observed a significant increase in the expression of transcripts for STRAP and NME1 in the mouse VM between E12 and E14, which is the period of maximal axonal outgrowth of mDA neurons toward their striatal targets. Consistent with this, NME1 is known to be expressed, and to interact with prune exopolyphosphatase (PRUNE)1, in the midbrain and SN of E14.5 mice (Carotenuto et al., 2006). We also report that STRAP and NME1 are both expressed in the SNpc of adult mice. Furthermore, we found that NME1 and TH were co-expressed in mDA neurons in the adult rat SN, further validating our gene co-expression data. Collectively, these data suggest that STRAP and NME1 play roles in the development and growth of nigrostriatal dopaminergic neurons.

In our study, the expression of both STRAP and NME1 exhibited strong positive correlation with that of TH, GIRK2, and ALDH1A1, all of which are markers of mDA neurons. Subsequently, we performed a gene ontology enrichment analysis of all the genes that are co-expressed with STRAP in the human SN. We observed that genes that were co-expressed with STRAP were involved in processes such as postsynaptic intermediate filament cytoskeleton organization and neurofilament cytoskeleton organization. We then obtained a list of all genes that are co-expressed with NME1 in the human SN and performed a GO analysis on these. We found that processes that were significantly enriched included circadian sleep/wake cycle and neurofilament cytoskeleton organization. Further analysis of those genes involved in neurofilament cytoskeleton organization in the human SN showed strong correlation between the expression of both NME1 and STRAO and that of several key proteins that are involved in cytoskeletal processes, namely, INA, NEFH, ATP8A2, NEFL, SOD1, and NudE NEDL1.

Together, these data suggest that NME1 and STRAP are involved in GDF5-mediated neurite growth. In agreement with this, we found that silencing of NME1 resulted in impaired neurite growth in SH-SH5Y cells, a process that is used as a marker of neural differentiation. Furthermore, treatment of SH-SH5Y cells with GDF5 had no effect on neurite growth when NME1 was silenced. Given these data, we propose that NME1 is an essential downstream effector of GDF5-induced enhancement of neurite growth. Moreover, we found that NME1 overexpression was sufficient to promote axon growth, and that treatment with recombinant NME1 protein significantly increased neurite outgrowth in both SH-SY5Y cells and cultured mDA neurons. In agreement with our data, several other studies have described a role for NME1 in neurite growth, in other cell types. For example, Wright and colleagues showed that treatment of a collagen substrate with recombinant NME1 resulted in increased neurite growth in both chick and rat dorsal root ganglial cell cultures (Wright et al., 2010). Moreover, it is known that NME1 is secreted and released in cerebrospinal fluid from patients with traumatic brain injury (Lescuyer et al., 2004) and in neurospheres cultured from mouse cerebral cortex (Lööv et al., 2013). To our knowledge, ours is the first report of neurite growth-promoting effects of NME1 in DA neurons. Collectively, our data suggest that GDF5-induced upregulation of NME1 is involved in the known neurite growth-promoting action of this neurotrophic factor.

We also found that silencing of STRAP impaired basal and GDF5-promoted neurite growth, and that STRAP overexpression promoted neurite growth in SH-SY5Y cells. To our knowledge, this is the first report of a function for STRAP in the regulation of axonal growth. A proteome analysis study found that STRAP protein levels are regulated during the differentiation of the human fetal midbrain stem cell line, ReNcell VM (Hoffrogge et al., 2006); this suggests that regulation of STRAP expression may be associated with cellular differentiation. It is well characterized that STRAP inhibits apoptosis by directly interacting with apoptosis signal-regulating kinase (ASK)1 (Jung et al., 2010). As there is increased apoptosis and axonal degeneration in the PD brain, GDF5-mediated upregulation of STRAP protein levels in the brain may be involved in the neuroprotective effects of this factor. Furthermore, it is known that STRAP interacts with TGFβ-interacting protein and that phosphorylation of STRAP at its Ser188 residue by serine/threonine kinase 38 (MPK38) is important for the pro-apoptotic function of STRAP (Seong et al., 2014). As many kinases are known to play roles in PD neuropathology, STRAP inhibition may provide a new therapeutic approach for slowing the progression of PD.

Our analysis of datasets of gene expression in PD and control SN found that the expression of NME1 was significantly lower in the PD brain. In addition, there was a loss of the normal co-expression pattern of NME1 with the mDA marker, ALDHA1A, in PD patient samples. It has been well-documented that normal gene co-expression patterns are disrupted in disease states, and that these broken correlations can be used as an index of functional misregulation (Torkamani et al., 2010; Zhang et al., 2013; Southworth et al., 2009). NME1 expression has also reported to be downregulated in Alzheimer disease (Cieślak and Wojtczak, 2018). GDF5-mediated increases in NME1 levels could potentially be applied to address the loss in NME1 expression, and to restore its co-expression profile in mDA neurons, with the aim of conferring neuroprotection in PD.

STRAP expression was slightly, although not significantly, reduced in the PD brain, whereas its co-expression with ALDHA1A was significantly impaired. Such loss of co-expression in PD may reflect a role for STRAP in endogenous protective mechanisms in the brain, which may be adversely affected by PD pathology. It is known that STRAP has antiapoptotic effects (Jung et al., 2010) and that it can regulate the heat shock protein (HSP) response by acting as a modulator of p300 (Xu et al., 2008). As HSP signaling is thought to be a crucial cytoprotective mechanism in PD (Luo et al., 2006), GDF5-mediated upregulation of STRAP might confer a neuroprotective effect, in addition to its role in increasing neurite growth. This loss of STRAP/ALDHA1A co-expression in PD could potentially be rescued by GDF5 treatment, which may be sufficient to restore levels of STRAP in mDA neurons.

Activation of the BMP-Smad pathway by the GDF5-related factor BMP2 has been found to restore neurite growth in 1-methyl-4-phenylpyridinium (MPP^+^)-, 6-OHDA- and α-synuclein-induced *in vitro* models of PD (Goulding et al., 2019). Delivery of neurotrophic proteins such as GDF5 and BMP2 to the PD brain is associated with issues due to their large size and rapid metabolism, thus strategies aimed at manipulation of their downstream signaling molecules would be advantageous. The discovery in the present study that GDF5 treatment induced increases in neurite growth through the regulation of NME1 and STRAP proteins rationalizes the further study of NME1 and STRAP as potential neuroprotective targets that may be useful for therapies aimed at axonal regeneration in PD.

### Limitations of the Study

In our current study, we used SH-SY5Y cells as a model of dopaminergic neurons rather than primary cultures of dopaminergic neurons. This was to ensure a homogeneous population of cells for proteome analysis, rather than mixed cell cultures of embryonic rat midbrain tissue. Furthermore, SH-SY5Y cells express several markers of dopaminergic neurons, and are a widely used *in vitro* model for the study of molecular signaling of relevance to PD.

### Resource Availability

#### Lead Contact

Further information and requests for resources and reagents should be directed to and will be fulfilled by the Lead Contact, Gerard O'Keeffe (g.okeeffe@ucc.ie).

#### Materials Availability

All unique/stable reagents generated in this study are available from the Lead Contact without restriction.

### Data and Code Availability

The raw data supporting the conclusions of this manuscript will be made available by the authors, without undue reservation, to any qualified researcher.

## Methods

All methods can be found in the accompanying Transparent Methods supplemental file.

## Supplemental Information

Document S1. Transparent Methods

## References

[bib1] Burke R.E., O'Malley K. (2013). Axon degeneration in Parkinson's disease. Exp. Neurol..

[bib2] Caminiti S.P., Presotto L., Baroncini D., Garibotto V., Moresco R.M., Gianolli L., Volonté M.A., Antonini A., Perani D. (2017). Axonal damage and loss of connectivity in nigrostriatal and mesolimbic dopamine pathways in early Parkinson's disease. Neuroimage. Clin..

[bib3] Carotenuto P., Marino N., Bello A.M., D'Angelo A., Di Porzio U., Lombardi D., Zollo M. (2006). PRUNE and NM23-M1 expression in embryonic and adult mouse brain. J. Bioenerg. Biomembr..

[bib4] Cieślak M., Wojtczak A. (2018). Role of purinergic receptors in the Alzheimer’s disease. Purinergic Signal..

[bib5] Costello D.J., O'Keeffe G.W., Hurley F.M., Sullivan A.M. (2012). Transplantation of novel human GDF5-expressing CHO cells is neuroprotective in models of Parkinson's disease. J. Cell Mol. Med..

[bib6] Decressac M., Kadkhodaei B., Mattsson B., Laguna A., Perlmann T., Björklund A. (2012). α-Synuclein-induced down-regulation of Nurr1 disrupts GDNF signaling in nigral dopamine neurons. Sci. Transl. Med..

[bib7] Dijkstra A.A., Ingrassia A., de Menezes R.X., van Kesteren R.E., Rozemuller A.J., Heutink P., van de Berg W.D. (2015). Evidence for immune response, axonal dysfunction and reduced endocytosis in the substantia nigra in early stage Parkinson's disease. PLoS One.

[bib8] Drinkut A., Tillack K., Meka D.P., Schulz J.B., Kügler S., Kramer E.R. (2016). Ret is essential to mediate GDNF's neuroprotective and neuroregenerative effect in a Parkinson disease mouse model. Cell Death Dis..

[bib9] Eisen M.B., Spellman P.T., Brown P.O., Botstein D. (1998). Cluster analysis and display of genome-wide expression patterns. Proc. Natl. Acad. Sci. U S A.

[bib10] Garcia-Esparcia P., Hernández-Ortega K., Ansoleaga B., Carmona M., Ferrer I. (2015). Purine metabolism gene deregulation in Parkinson's disease. Neuropathol. Appl. Neurobiol..

[bib11] Gavin A.M., Walsh S., Wyatt S., O'Keeffe G.W., Sullivan A.M. (2014). 6-Hydroxydopamine induces distinct alterations in GDF5 and GDNF mRNA expression in the rat nigrostriatal system in vivo. Neurosci. Lett..

[bib12] Gill S.S., Patel N.K., Hotton G.R., O'Sullivan K., McCarter R., Bunnage M., Brooks D.J., Svendsen C.N., Heywood P. (2003). Direct brain infusion of glial cell line-derived neurotrophic factor in Parkinson disease. Nat. Med..

[bib13] Goulding S.R., Sullivan A.M., O'Keeffe G.W., Collins L.M. (2019). Gene co-expression analysis of the human substantia nigra identifies BMP2 as a neurotrophic factor that can promote neurite growth in cells overexpressing wild-type or A53T α-synuclein. Parkinsonism Relat. Disord..

[bib14] Hegarty S.V., Sullivan A.M., O'Keeffe G.W. (2013). BMP2 and GDF5 induce neuronal differentiation through a Smad dependant pathway in a model of human midbrain dopaminergic neurons. Mol. Cell Neurosci..

[bib15] Hegarty S.V., Collins L.M., Gavin A.M., Roche S.L., Wyatt S.L., Sullivan A.M., O'Keeffe G.W. (2014). Canonical BMP-Smad signalling promotes neurite growth in rat midbrain dopaminergic neurons. Neuromolecular Med..

[bib16] Hegarty S.V., Sullivan A.M., O’Keeffe G.W. (2018). Inhibition of miR-181a promotes midbrain neuronal growth through a Smad1/5-dependent mechanism: implications for Parkinson’s disease. Neuronal Signal..

[bib17] Hegarty S.V., Wyatt S.L., Howard L., Stappers E., Huylebroeck D., Sullivan A.M., O'Keeffe G.W. (2017). Zeb2 is a negative regulator of midbrain dopaminergic axon growth and target innervation. Sci. Rep..

[bib18] Hoffrogge R., Mikkat S., Scharf C., Beyer S., Christoph H., Pahnke J., Mix E., Berth M., Uhrmacher A., Zubrzycki I.Z. (2006). 2-DE proteome analysis of a proliferating and differentiating human neuronal stem cell line (ReNcell VM). Proteomics.

[bib19] Homouz D., Kudlicki A.S. (2013). The 3D organization of the yeast genome correlates with co-expression and reflects functional relations between genes. PLoS One.

[bib20] Hurley F.M., Costello D.J., Sullivan A.M. (2004). Neuroprotective effects of delayed administration of growth/differentiation factor-5 in the partial lesion model of Parkinson's disease. Exp. Neurol..

[bib21] Jaumotte J.D., Zigmond M.J. (2014). Comparison of GDF5 and GDNF as neuroprotective factors for postnatal dopamine neurons in ventral mesencephalic cultures. J. Neurosci. Res..

[bib22] Jung H., Seong H.-A., Manoharan R., Ha H. (2010). Serine-threonine kinase receptor-associated protein inhibits apoptosis signal-regulating kinase 1 function through direct interaction. J. Biol. Chem..

[bib24] Kelly M.J., O'Keeffe G.W., Sullivan A.M. (2015). Viral vector delivery of neurotrophic factors for Parkinson's disease therapy. Expert Rev. Mol. Med..

[bib25] Krieglstein K., Unsicker K. (1995). Bovine chromaffin cells release a transforming growth factor-β-like molecule contained within chromaffin granules. J. Neurochem..

[bib26] Lang A.E., Gill S., Patel N.K., Lozano A., Nutt J.G., Penn R., Brooks D.J., Hotton G., Moro E., Heywood P. (2006). Randomized controlled trial of intraputamenal glial cell line–derived neurotrophic factor infusion in Parkinson disease. Ann. Neurol..

[bib27] Lees A.J., Hardy J., Revesz T. (2009). Parkinson's disease. Lancet.

[bib28] Lescuyer P., Allard L., Zimmermann-Ivol C.G., Burgess J.A., Hughes-Frutiger S., Burkhard P.R., Sanchez J.-C., Hochstrasser D.F. (2004). Identification of post-mortem cerebrospinal fluid proteins as potential biomarkers of ischemia and neurodegeneration. Proteomics.

[bib29] Liu J., Saito K., Maruya Y., Nakamura T., Yamada A., Fukumoto E., Ishikawa M., Iwamoto T., Miyazaki K., Yoshizaki K. (2016). Mutant GDF5 enhances ameloblast differentiation via accelerated BMP2-induced Smad1/5/8 phosphorylation. Sci. Rep..

[bib30] Lööv C., Shevchenko G., Geeyarpuram Nadadhur A., Clausen F., Hillered L., Wetterhall M., Erlandsson A. (2013). Identification of injury specific proteins in a cell culture model of traumatic brain injury. PLoS One.

[bib31] Luo G.R., Chen S., Le W.D. (2006). Are heat shock proteins therapeutic target for Parkinson's disease?. Int. J. Biol. Sci..

[bib32] Macpherson P.C.D., Farshi P., Goldman D. (2015). Dach2-Hdac9 signaling regulates reinnervation of muscle endplates. Development.

[bib33] Makkar P., Metpally R.P., Sangadala S., Reddy B.V. (2009). Modeling and analysis of MH1 domain of Smads and their interaction with promoter DNA sequence motif. J. Mol. Graph. Model..

[bib34] Marks W.J., Bartus R.T., Siffert J., Davis C.S., Lozano A., Boulis N., Vitek J., Stacy M., Turner D., Verhagen L. (2010). Gene delivery of AAV2-neurturin for Parkinson's disease: a double-blind, randomised, controlled trial. Lancet Neurol..

[bib35] Marks W.J., Ostrem J.L., Verhagen L., Starr P.A., Larson P.S., Bakay R.A., Taylor R., Cahn-Weiner D.A., Stoessl A.J., Olanow C.W., Bartus R.T. (2008). Safety and tolerability of intraputaminal delivery of CERE-120 (adeno-associated virus serotype 2-neurturin) to patients with idiopathic Parkinson's disease: an open-label, phase I trial. Lancet Neurol..

[bib36] Meister G., Bühler D., Pillai R., Lottspeich F., Fischer U. (2001). A multiprotein complex mediates the ATP-dependent assembly of spliceosomal U snRNPs. Nat. Cell Biol..

[bib37] O'Keeffe G.W., Dockery P., Sullivan A.M. (2004). Effects of growth/differentiation factor 5 on the survival and morphology of embryonic rat midbrain dopaminergic neurones in vitro. J. Neurocytol..

[bib39] O’Keeffe G.W., Gutierrez H., Howard L., Laurie C.W., Osorio C., Gavaldà N., Wyatt S.L., Davies A.M. (2016). Region-specific role of growth differentiation factor-5 in the establishment of sympathetic innervation. Neural Dev..

[bib38] O'Keeffe G.W., Hanke M., Pohl J., Sullivan A.M. (2004). Expression of growth differentiation factor-5 in the developing and adult rat brain. Brain Res. Dev. Brain Res..

[bib40] O’Keeffe G.W., Hegarty S.V., Sullivan A.M. (2017). Targeting bone morphogenetic protein signalling in midbrain dopaminergic neurons as a therapeutic approach in Parkinson’s disease. Neuronal Signal..

[bib41] O'Keeffe G.W., Sullivan A.M. (2018). Evidence for dopaminergic axonal degeneration as an early pathological process in Parkinson's disease. Parkinsonism Relat. Disord..

[bib42] Osorio C., Chacon P.J., Kisiswa L., White M., Wyatt S., Rodriguez-Tebar A., Davies A.M. (2013). Growth differentiation factor 5 is a key physiological regulator of dendrite growth during development. Development.

[bib43] Owlanj H., Jie Yang H., Wei Feng Z. (2012). Nucleoside diphosphate kinase Nm23-M1 involves in oligodendroglial versus neuronal cell fate decision in vitro. Differentiation.

[bib44] Paul G., Sullivan A.M. (2018). Trophic factors for Parkinson's disease: where are we and where do we go from here?. Eur. J. Neurosci..

[bib45] Ramasamy A., Trabzuni D., Guelfi S., Varghese V., Smith C., Walker R., De T., Consortium U.K.B.E., North American Brain Expression C., Coin L. (2014). Genetic variability in the regulation of gene expression in ten regions of the human brain. Nat. Neurosci..

[bib46] Seong H.A., Jung H., Choi H.S., Kim K.T., Ha H. (2005). Regulation of transforming growth factor-beta signaling and PDK1 kinase activity by physical interaction between PDK1 and serine-threonine kinase receptor-associated protein. J. Biol. Chem..

[bib47] Seong H.A., Jung H., Ha H. (2007). NM23-H1 tumor suppressor physically interacts with serine-threonine kinase receptor-associated protein, a transforming growth factor-beta (TGF-beta) receptor-interacting protein, and negatively regulates TGF-beta signaling. J. Biol. Chem..

[bib48] Seong H.A., Manoharan R., Ha H. (2014). A crucial role for the phosphorylation of STRAP at Ser(188) by MPK38 in STRAP-dependent cell death through ASK1, TGF-β, p53, and PI3K/PDK1 signaling pathways. Cell Cycle.

[bib49] Sharma A., Lambrechts A., Hao L.t., Le T.T., Sewry C.A., Ampe C., Burghes A.H.M., Morris G.E. (2005). A role for complexes of survival of motor neurons (SMN) protein with gemins and profilin in neurite-like cytoplasmic extensions of cultured nerve cells. Exp. Cell Res..

[bib50] Slevin J.T., Gash D.M., Smith C.D., Gerhardt G.A., Kryscio R., Chebrolu H., Walton A., Wagner R., Young A.B. (2007). Unilateral intraputamenal glial cell line-derived neurotrophic factor in patients with Parkinson disease: response to 1 year of treatment and 1 year of withdrawal. J. Neurosurg..

[bib51] Southworth L.K., Owen A.B., Kim S.K. (2009). Aging mice show a decreasing correlation of gene expression within genetic modules. PLoS Genet..

[bib52] Storm E.E., Huynh T.V., Copeland N.G., Jenkins N.A., Kingsley D.M., Lee S.J. (1994). Limb alterations in brachypodism mice due to mutations in a new member of the TGF beta-superfamily. Nature.

[bib53] Storm E.E., Kingsley D.M. (1996). Joint patterning defects caused by single and double mutations in members of the bone morphogenetic protein (BMP) family. Development.

[bib54] Sullivan A.M., Opacka-Juffry J., Hotten G., Pohl J., Blunt S.B. (1997). Growth/differentiation factor 5 protects nigrostriatal dopaminergic neurones in a rat model of Parkinson’s disease. Neurosci. Lett..

[bib55] Sullivan A.M., Pohl J., Blunt S.B. (1998). Growth/differentiation factor 5 and glial cell line-derived neurotrophic factor enhance survival and function of dopaminergic grafts in a rat model of Parkinson's disease. Eur. J. Neurosci..

[bib56] Sullivan A.M., O'Keeffe G.W. (2016). Neurotrophic factor therapy for Parkinson's disease: past, present and future. Neural Regen. Res..

[bib57] Torkamani A., Dean B., Schork N.J., Thomas E.A. (2010). Coexpression network analysis of neural tissue reveals perturbations in developmental processes in schizophrenia. Genome Res..

[bib58] Toulouse A., Collins G.C., Sullivan A.M. (2012). Neurotrophic effects of growth/differentiation factor 5 in a neuronal cell line. Neurotoxicity Res..

[bib59] Tysnes O.-B., Storstein A. (2017). Epidemiology of Parkinson’s disease. J. Neural Transm..

[bib60] Warren Olanow C., Bartus R.T., Baumann T.L., Factor S., Boulis N., Stacy M., Turner D.A., Marks W., Larson P., Starr P.A. (2015). Gene delivery of neurturin to putamen and substantia nigra in Parkinson disease: a double-blind, randomized, controlled trial. Ann. Neurol..

[bib61] Whone A., Luz M., Boca M., Woolley M., Mooney L., Dharia S., Broadfoot J., Cronin D., Schroers C., Barua N.U. (2019). Randomized trial of intermittent intraputamenal glial cell line-derived neurotrophic factor in Parkinson's disease. Brain.

[bib62] Wright K.T., Seabright R., Logan A., Lilly A.J., Khanim F., Bunce C.M., Johnson W.E.B. (2010). Extracellular Nm23H1 stimulates neurite outgrowth from dorsal root ganglia neurons in vitro independently of nerve growth factor supplementation or its nucleoside diphosphate kinase activity. Biochem. Biophys. Res. Commun..

[bib63] Xicoy H., Wieringa B., Martens G.J.M. (2017). The SH-SY5Y cell line in Parkinson’s disease research: a systematic review. Mol. Neurodegener..

[bib64] Xu D., Zalmas L.P., La Thangue N.B. (2008). A transcription cofactor required for the heat-shock response. EMBO Rep..

[bib65] Zhang B., Gaiteri C., Bodea L.G., Wang Z., McElwee J., Podtelezhnikov A.A., Zhang C., Xie T., Tran L., Dobrin R. (2013). Integrated systems approach identifies genetic nodes and networks in late-onset Alzheimer's disease. Cell.

