# Supplementary material for: STRAP and NME1 Mediate the Neurite Growth-Promoting Effects of the Neurotrophic Factor GDF5
Source: iScience. 2020 Aug 12;23(9):101457. doi: 10.1016/j.isci.2020.101457 (PMC7452236; doi:10.1016/j.isci.2020.101457)
Supplement: Document S1. Transparent Methods [file mmc1.pdf]

iScience, Volume *23*

Supplemental Information

STRAP and NME1 Mediate the Neurite

Growth-Promoting Effects

# of the Neurotrophic Factor GDF5

Jayanth Anantha, Susan R. Goulding, Sean L. Wyatt, Ruth M. Concannon, Louise M. Collins, Aideen M. Sullivan, and Gerard W. O'Keeffe

SUPPLEMENTAL INFORMATION.

TRANSPARENT METHODS

Cell culture and treatment. SH-SY5Y cells were grown in DMEM-high glucose (D5796) supplemented with 10% (v/v) FBS, 1% (v/v) glutamine (G7513), 1% (v/v) non-essential amino acids (M7145), 1% (v/v) sodium pyruvate (S8636), and 1% (v/v) penicillin/streptomycin (P4333) (all from Sigma). Cells were cultured at 37°C with 5% CO_2. For Western blotting and RNA extraction, 2.5x10^6 SH-SY5Y cells were plated overnight on a 6-well plate and treated with 100ng/ml of GDF5 (Preprotech; Cat no.120-01) for 0 to 240 min. For analysis of neurite length, 30,000-50,000 SH-SY5Y cells were plated overnight on a 24-well plate and treated with 100 ng/ml GDF5 for 72h, or with 10, 50, 100 or 200 ng/ml of recombinant human NME1 (Novus Biological; Cat no. NBP2-252250) for 48 h. Primary cultures of E14 rat VM were prepared as described in Hegarty et al., 2014. In brief, E14 embryos were obtained from date-mated Sprague-Dawley rats under terminal anaesthesia using isoflurane. Dissected VM tissue was centrifuged at 1100 rpm for 5 min at 4˚C. The tissue pellet was incubated in 0.1% trypsin-Hank's Balanced Salts solution for 5 min at 37˚C with 5% CO_2. Following addition of fetal calf serum and centrifugation at 1100 rpm for 5 min at 4˚C, the resulting cell pellet was resuspended in 1 ml of differentiation media (Dulbecco's modified Eagle's medium/F12, 33 mM D-glucose, 1% L-glutamine, 1% FCS, supplemented with 2% B27) and triturated. Cells were plated on poly-D-lysine (Sigma)-coated 24-well tissue culture plates at a density of 5 x 10^4 cells per well in 500 μl of differentiation media at 37˚C with 5% CO_2. Cultures were treated with 100 ng/ml recombinant human NME1 for 48 h.

Western blotting. Cells were lysed in RIPA buffer (10 mM Tris-Cl (pH 8.0), 1 mM EDTA, 0.5 mM EGTA, 1% Triton X-100, 0.1% sodium deoxycholate, 0.1% SDS, 140 mM NaCl) supplemented with 1 mM sodium orthovanadate, 1mM sodium fluoride and complete protease inhibitor cocktail, as per the manufacturer's recommendation (Roche Molecular Biochemicals; 11836170001) for 30 min on ice. Lysates were centrifuged at 13,000 rpm for 20 min at 4°C and the supernatant was transferred into a fresh tube. The protein concentration of the samples was determined using the BCA method (Pierce; Cat no. 23227) and stored at -20°C prior to analysis. Upon use, lysates were mixed with a 1x sample loading buffer (SLB) (5xSLB-containing 70ml glycerol 30ml water with 0.2g of bromophenol blue, 2.5g sodium dodecyl sulphate, 0.606g of Tris base with 5-6% v/v β-mercaptoethanol) and boiled at 95°C for 5-10 min. The samples were then resolved on a SDS-PAGE gel and transferred to a PVDF membrane. The membrane was blocked in blocking buffer (containing 5% BSA and 0.1% Tween20 in 1xPBS/1xTBS) for 1 h at room temperature, then sequentially probed with primary and the appropriate HRP-linked secondary antibodies made up in blocking buffer for 1h at room temperature. Enhanced Chemi-Luminescence (ECL) was performed using an ECL kit (Thermo Scientific; Cat-32106) following detection and development on an X-ray film for optimised durations of exposure in a dark room. The films were then scanned and analysed by densitometry using ImageJ software.

Immunocytochemical staining. SH-SY5Y cells were fixed in 4% PFA at 4°C for 20 min at room temperature. The cells were then washed with 10mM PBS and permeablized for 30 min with 10mM PBS containing 0.01% Triton X-100. The cells were then blocked in 1% BSA made in 10mM PBS containing 0.01% Triton X-100 for 1 h at room temperature, and then washed in 10mM PBS. Cell were then immunostained with primary antibodies (see below) diluted in 1% BSA-PBS containing 0.01% Triton X-100 for 12 h at 4°C, washed with 10mM PBS and then probed with secondary antibodies diluted 1% BSA-PBS containing 0.01% Triton X-100 for 1 h at room temperature. Nuclei were counter stained using DAPI at a concentration of 0.1µg/ml in 10mM PBS for one min. Following washes with 10mM PBS, imaging was carried out with an Olympus IX71 inverted microscope fitted with an Olympus DP70 camera and the resultant images were analysed using ImageJ software.

Antibodies. The following antibodies were used for Western blotting and immunocytochemical staining. Anti-GAPDH (SCBT; SC-47724, 1:1000(WB), 1:200(IF)); Anti-Smad1/5/9 (Abcam; ab66737, 1:1000(WB), 1:200(IF)); Anti-p-Smad1/5/9 (CST; 13820, 1:1000(WB), 1:200(IF)). HRP-conjugated anti-mouse (ThermoFisher; Cat-A27025) was used at 1:5000 and anti-rabbit ThermoFisher; Cat-31460) was used at 1:5000. Alexa Fluor-488 anti-rabbit and Alexa Fluor-594 anti-mouse conjugated antibodies were used at a concentration of 1:500 (Invitrogen).

qRT-PCR. Cells were removed from the tissue culture dish using a sterile cell scraper and centrifuged at 1500 rpm for 5 min at 4°C. Subsequently, the pellets were lysed in 500µl of Tri reagent and 250µl Chloroform, vortexed and centrifuged at 13,200 rpm for 10 min. The aqueous layer was separated into an Eppendorf tube and 500µl of isopropanol and vortexed briefly and centrifuged at 13,200 rpm for 15 min. The supernatant was discarded and the pellets were washed with 80% ethanol and re-pelleted by centrifugation at 13,200 rpm for 10 min, subsequently air dried for 30 min and dissolved in RNase free water. The RNA yield and purity were measured on a Nano drop with nuclease free water as blank. cDNA was synthesized using random hexamers. A High capacity cDNA reverse transcription kit (Life Technologies; cat. no 4368814) was used as per the manufacturers recomendations. The synthesized c-DNA was used for real time PCR using the Jump start SYBR green kit (Sigma; cat no. S4438). The primers used were as follows *Hes5* FW: 5’ CGCATCAACAGCAGCATCGAG 3’ RV: 5’ GACGAAGGCTTTGCTGTGCT 3’, *Zeb2* FW: 5’ CGACACTCTTGGCGAGGTTT 3’ RV: 5’ TCACCACTGCGAAGTCTTGTT 3’ and *Gapdh* FW: 5’ CCACTAGGCGCTCACTGTT 3’ RV: 5’ ACCAGAGTTAAAAGCAGCCC 3’. RT-PCR was performed on a Real time PCR machine by (Life echnologies; cat no. 4376600). The data was then analysed by δδ-Ct method; values for individual genes calculated against an endogenous control.

To profile mRNA expression in the developing mouse VM, the levels of *Strap* and *Nme1* mRNAs were quantified by real-time PCR relative to a geometric mean of mRNAs for the house keeping enzymes glyceraldehyde phosphate dehydrogenase (*Gapdh*), succinate dehydrogenase (*Sdha*) and hypoxanthine phosphoribosyltransferase-1 (*Hprt1*). Total RNA was extracted from dissected VM using the RNeasy lipid mini extraction kit (Qiagen, UK) and 5 µl was reverse transcribed for 1 h at 45°C using the AffinityScript kit (Agilent, UK) in a 25 µl

reaction according to the manufacturer's instructions. 2 µl of cDNA was amplified in a 20 µl reaction volume using Brilliant III ultrafast qPCR master mix reagents (Agilent). PCR products were detected using dual-labeled (FAM/BHQ1) hybridization probes specific to each of the cDNAs (MWG/Eurofins, Germany). The PCR primers were: *Strap* forward: 5'-CGGGTGGAGAAGACTTTA-3' and reverse: 5'-CACAGTTTGCCACAATCTC-3'; *Nme1* forward: 5'-TCTCCCTTCCTATCACCTG-3' and reverse: 5'-CACACATCCTCCACACAA-3'; *Gapdh* forward: 5'-GAGAAACCTGCCAAGTATG-3' and reverse: 5'-GGAGTTGCTGTTGAAGTC-3'; *Sdha* forward: 5'-GGAACACTCCAAAAACAG-3' and reverse: 5'-CCACAGCATCAAATTCAT-3'; *Hprt1* forward: 5'-TTAAGCA GTACAGCCCCAAAATG-3' and reverse: 5'-AAGTCTGGCCTG TATCCAACAC-3'. Dual-labeled probes were: *Strap*: 5'-FAM-TGTCCCATCTTCAGAACCGCT-BHQ1-3'; *Nme1*: 5'-FAM-TCCTGGCACAGTCAGACAACA-BHQ1-3'; *Gapdh*: 5'-FAM-AGACAACCTG GTCCTCAGTGT-BHQ1-3; *Sdha*: 5′-FAM-CCTGCGGCTTTCACTTCTCT-BHQ1-3', *Hrpt1*: 5'-FAM-TCGAGAGGTCCTTTTCACCAGCAAG-BHQ1-3'. Forward and reverse primers were used at a concentration of 150 nM and dual-labeled probes were used at a concentration of 300 nM. PCR was performed using the Mx3000P platform (Agilent) using the following conditions: 45 cycles of 95°C for 10 s and 60°C for 35 s. Standard curves were generated for each cDNA for every real time PCR run, by using serial five-fold dilutions of reverse-transcribed mouse adult brain total RNA (Zyagen, USA). Relative mRNA levels were quantified in four separate sets of dissected tissues for each age. Primer and probe sequences were designed using Beacon Designer software (Premier Biosoft, USA).

Gene Expression Analysis of the Human SN and mouse SN. Human SN gene expression data from healthy controls (GSE:60863) (Kang et al., 2011), and from PD and control subjects (GSE:49036) (Dijkstra et al., 2015), were analysed using the R2: Genomics Analysis and Visualization Platform (https://hgserver1.amc.nl/cgi-bin/r2/main.cgi). Pearson correlation analysis with a Bonferroni multiple testing correction was used to identify those genes with a significant correlation with *STRAP* and *NME1* expression, and to examine *STRAP-NME1* co-expression. All gene expression data were log2 expression values. Gene ontology (GO) enrichment analysis was performed at www.geneontology.org.

Proteomics using label-free LC-MS/MS. SHSY5Y cells were treated with 100 ng/ml GDF5 for 240 min and lysed in RIPA buffer supplemented with 1 mM sodium orthovanadate, 1mM sodium fluoride and complete protease inhibitor cocktail (Roche Molecular Biochemicals) for 30 min on ice. Lysates were centrifuged at 13,200 rpm for 20 min. Supernatants were resolved on an SDS-Page gel and the gel was cut and shipped to Fingerprint Proteomics (University of Dundee, UK). Proteins were extracted from the gel, followed by trypsin digestion and passage through an Ultima 3000 nano-LC machine for analysis with a flow duration of 2 h. The raw data files were then analysed using MaxQuant version 1.6.0.16 carbamidomethylation as a fixed modification, all standard contaminants were filtered, 1% FDR was used for peptide identification and LFQ intensities were procured, analysis was performed with match between runs. From the data procured from MaxQuant, the average LFQ intensities and the fold change ratios were calculated. A two tailed Paired Student's t-test was performed to identify

significantly upregulated proteins. -Log 10 of P-values and Log2 of fold change were calculated and volcano plots were generated using the R-script EnhancedVolcano version 1.4.0 https://github.com/kevinblighe/EnhancedVolcano) (R version 3.5.3) .

Transfections. SH-SY5Y cells were plated in 24-wells plates and allowed to grow overnight. Transfections were performed using TransIT-X2® reagent following the manufacturers guidelines. For transfections with DsiRNA, 25nM of siRNA of the following double-stranded siRNAs: Scrambled (siSCR) (FW: 5'-CGUUAAUCGCGUAUAAUACGCGUAT-3': 5'-AUACGCGUAUUAUACGCGAUUAACGAC-3'), siNME1 (FW: 5'-GAGGACUGGUAGAUUACACGAGCT-3' RV-5'-AGCUCGUGUAAUCUACCAGUUCCUCAG-3') and siSTRAP (FW: 5'-AGGAUAAACUGUUACGCAUAUAUGA-3' RV-5'-UCAUAUUGCGUAACAGUUUAUCCUGU-3'). A STRAP-expressing plasmid was purchased from Origene (RC209149) and the vector backbone was pCMV6 with a CMV promoter, followed by an N-terminal Myc-DDk tag on the STRAP protein. FLAG-NM23-H1 was a gift from Judy Lieberman (Addgene plasmid #25000; http://n2t.net/addgene:25000; RRID: Addgene_25000) and the NME1/NM23 construct had an N-terminal Flag tag followed by the NM23 coding sequence in a pCMV4 vector. In all cases, cells were co-transfected with an eGFP-expressing plasmid to identify transfected cells. Where indicated, cultures were treated with 100 ng/ml GDF5 daily for 72 h.

Neurite length analysis. Transfection and treatments were performed in duplicates as described above. Cells were imaged using an Olympus IX71 inverted microscope with the CellSense suit. Five images were captured from each well and neurite length was analysed using Image J. The averages of all measurements from each independent experiment were obtained and used to compare differences in the length of neurites between groups.

Animal Husbandry. Adult female Sprague- Dawley rats were procured from Envigo, UK, and maintained on a 12h:12h light:dark cycle with access to food and water *ad libitum.* Rats were housed in groups of four in standard housing cages containing environmental enrichment. All experiments were conducted fully in accordance with the European Directive 2010/63/EU and under an authorisation granted by the Health Products Regulatory Authority Ireland (AE19130/P057).

Virus Preparation and Stereotactic Surgery. AAV2/5-GDF5, AAV2/5-Null and AAV2/6-Null viral vectors were produced by Vector Biosystems Inc, Philadelphia, USA. In brief, AAV2 inverted terminal repeats coding for human GDF5 were packaged using AAV6 or AAV5 capsid proteins, to produce AAV2/6 and AAV2/5 viral vectors and the corresponding empty control vectors. Transgene expression was driven by synapsin- 1 promoter and enhanced using woodchuck hepatitis virus post- transcriptional regulatory element (WPRE). Stereotactic surgery was conducted under general anaesthesia induced by the inhalation agent isoflurane. Each animal was placed in a stereotactic frame, an incision was made to the scalp and a small hole was drilled into the skull. AAV-Control animals were administered 2 µL AAV2/5-Null (1.0×10^{10} vg/µL) + AAV2/6-Null (5.3×10^{9} vg/µL). AAV-GDF5 animals received 2 µL AAV2/5-GDF5 (1.3×10^{10} vg/µL) + AAV2/6-Null (5.3×10^{9} vg/µL). All vector

combinations were given unilaterally into the SN at coordinates AP - 5.3, ML ± 2.0, DV - 7.2 relative to bregma, at an infusion rate of 1µl/min with an additional 2 min for diffusion, before withdrawal and suturing. Post-surgery, animals received the analgesic Carprofen (5 mg/kg, s.c.) and 5% glucose solution (i.p.) and were allowed to recover fully on a heating-mat before being returned to their home cages.

Tissue Processing. Animals were sacrificed 20 weeks post-surgery by transcardial perfusion-fixation under terminal pentobarbital anaesthesia (50 mg/kg) for immunohistochemical analyses. Brains were post-fixed in 4% paraformaldehyde for 24 h and cryoprotected in 30% sucrose solution with 0.1% sodium azide. Sections (30 µm thickness) were cut on a freezing stage sledge microtome and were used for immunohistochemistry.

Immunohistochemical staining. Coronal sections through the SN were mounted onto gelatine-coated slides and washed for 10 min in tris-buffered saline (TBS) solution. Non-specific antibody binding was blocked for 1 h using 3% goat serum diluted in TBS containing 0.02% Triton-X100 (TXTBS). Sections were incubated overnight at room temperature with primary antibody diluted in TXTBS containing 1% goat serum. Primary antibodies used were: TH (Merck Millipore; 1:500) and NME1 (Cell Signaling Technologies; 1:100). Following 3 x 5-min TBS washes, sections were incubated for 2 h in Alexa Fluor® 594 goat-anti-mouse IgG (Invitrogen; 1:200, and Alexa Fluor® 488 goat-anti-rabbit IgG (Invitrogen; 1:200) secondary antibodies diluted in TXTBS containing 1% goat serum. Sections were washed for 3 x 5-min using TBS and cover-slipped using fluorescent mounting media (Dako Diagnostics). Images were taken using the Olympus FV1000 Confocal Laser Scanning Biological Microscope.

Statistical methods. Statistical analyses was performed using GraphPad Prism version 6 (©2018 GraphPad software, CA USA). Students's *t*-test, or one-way or two-way ANOVA with *post-hoc* tests as indicated in the Figure legends, were used as appropriate to identify statistically significant differences. All experiments were repeated independently at least three times.
